# Characterization of Regional Left Ventricular Function in Nonhuman Primates Using Magnetic Resonance Imaging Biomarkers: A Test-Retest Repeatability and Inter-Subject Variability Study

**DOI:** 10.1371/journal.pone.0127947

**Published:** 2015-05-26

**Authors:** Smita Sampath, Michael Klimas, Dai Feng, Richard Baumgartner, Elaine Manigbas, Ai-Leng Liang, Jeffrey L. Evelhoch, Chih-Liang Chin

**Affiliations:** 1 Imaging, Merck Research Laboratories, Merck Sharp & Dohme, Singapore, Singapore; 2 Imaging, Merck Research Laboratories, Merck & Co. Inc., West Point, Pennsylvania, United States of America; 3 Biometric Research, Merck Research Laboratories, Biostatistics and Research Decision Sciences, Merck & Co. Inc., Rahway, New Jersey, United States of America; 4 MRI Department, Maccine Pte. Ltd., Singapore, Singapore; University of Minnesota, UNITED STATES

## Abstract

Pre-clinical animal models are important to study the fundamental biological and functional mechanisms involved in the longitudinal evolution of heart failure (HF). Particularly, large animal models, like nonhuman primates (NHPs), that possess greater physiological, biochemical, and phylogenetic similarity to humans are gaining interest. To assess the translatability of these models into human diseases, imaging biomarkers play a significant role in non-invasive phenotyping, prediction of downstream remodeling, and evaluation of novel experimental therapeutics. This paper sheds insight into NHP cardiac function through the quantification of magnetic resonance (MR) imaging biomarkers that comprehensively characterize the spatiotemporal dynamics of left ventricular (LV) systolic pumping and LV diastolic relaxation. MR tagging and phase contrast (PC) imaging were used to quantify NHP cardiac strain and flow. Temporal inter-relationships between rotational mechanics, myocardial strain and LV chamber flow are presented, and functional biomarkers are evaluated through test-retest repeatability and inter subject variability analyses. The temporal trends observed in strain and flow was similar to published data in humans. Our results indicate a dominant dimension based pumping during early systole, followed by a torsion dominant pumping action during late systole. Early diastole is characterized by close to 65% of untwist, the remainder of which likely contributes to efficient filling during atrial kick. Our data reveal that moderate to good intra-subject repeatability was observed for peak strain, strain-rates, E/circumferential strain-rate (CSR) ratio, E/longitudinal strain-rate (LSR) ratio, and deceleration time. The inter-subject variability was high for strain dyssynchrony, diastolic strain-rates, peak torsion and peak untwist rate. We have successfully characterized cardiac function in NHPs using MR imaging. Peak strain, average systolic strain-rate, diastolic E/CSR and E/LSR ratios, and deceleration time were identified as robust biomarkers that could potentially be applied to future pre-clinical drug studies.

## Introduction

The complicated multifaceted pathogenesis of heart failure (HF) is still not fully understood. Over the past few decades, the rodent-to-man translational approach has shed insight into some fundamental biological and molecular mechanisms involved in HF and led to the recognition of specific molecular signaling pathways involved during the longitudinal cardiac remodeling process that results in HF [1]. Pioneering work conducted in various surgical as well as transgenic/knockout rodent models of HF has not only afforded an improved understanding of the relationships among myocardial injury, myocardial stress and remodeling [2,3], but has also contributed to the early exploration and evaluation of critical clinical treatment strategies such as angiotensin–converting enzyme inhibitor therapy [4,5], angiotensin II type 1 receptor antagonist therapy [6,7], and gene-regulated therapy [8,9,10]. Despite the advances gained from small animal studies, there exist significant differences between rodents and humans in cardiac characteristics such as heart rate, oxygen consumption, perfusion, metabolism, contractile protein expression, stem cell phenotypes and responses to protein alteration [11,12]. As a result, direct extrapolation of several findings in rodents has not been successful in the clinical setting. Large animal models that recapitulate the clinical phenotypes thus play a major role in bridging the basic science discoveries made in rodent models with clinical interventions and therapies. For example, landmark studies in surgical canine models have provided a deeper understanding of the spatial progression of myocardial ischemia, the time-course leading to eventual cell death and infarct expansion, and the discovery of achieving myocardial salvage through timely thrombolytic therapy [13,14]. However, the presence of significant collateral circulation in canine models alters their post injury remodeling in comparison with humans [15]. Porcine and ovine models of HF have been considered as superior alternatives to canine models and have successfully been used to produce more reproducible surgical models of myocardial injury and pressure overload [16,17]. Studies conducted in large animal models have also evaluated the upward translation of pharmacological [18,19,20], stem cell [21,22] or genetic therapy [23,24], previously tested in the rodent space, thus setting up the stage for future clinical translation.

Nonhuman primates (NHPs) are increasingly becoming the preferred non-rodent species for neuroscience and immunology research and have been widely used for drug discovery due to their physiological, biochemical, and phylogenetic similarity to humans [25,26]. Furthermore, studies indicate that in contrast to other large animal species, NHPs better reproduce metabolic phenotypes related to insulin resistance similar to human diabetics [27]. Additionally, the close histopathological similarity of lipids, pancreatic islets and lipoproteins in NHPs to humans and their propensity like humans to naturally develop diabetes when exposed to metabolic changes make them an ideal species to study cardiovascular interventions with metabolic comorbidities [28,29]. These findings have led to interest in the development of NHP models of ischemic and pressure-overload HF [30,31]. The characterization of NHP cardiac behavior in general and cardiac imaging biomarkers in specific is however grossly lacking in the literature. In particular, the identification of robust non-invasive functional imaging biomarkers for sensitive early assessment of NHP cardiac remodeling are critical to advancing NHP model development and drug safety/efficacy research related to cardiac diseases and still remains to be studied. Perhaps, more importantly, these imaging biomarkers can be further exploited to determine imaging phenotypes in NHP models of HF, which might afford translatable endpoints for clinical investigation of therapeutic responses.

This paper sheds insight into NHP cardiac function through the quantification of magnetic resonance (MR) imaging biomarkers that comprehensively characterize the regional and temporal behavior of left ventricular (LV) systolic pumping and LV diastolic relaxation. To our knowledge, there have been no published data describing regional cardiac function in NHPs. Herein, regional quantification of the spatial and temporal behavior of LV regional mechanics (strain and torsion) and LV chamber blood velocity in a cohort of eight naïve cynomolgus macaques is presented. The inter-relationships between NHP cardiac mechanics and hemodynamics are also presented. Specifically, spatial modulation of magnetization (SPAMM) tagged MR imaging [32,33] combined with harmonic phase (HARP) image analysis [34,35] was used to quantify strain and torsion, while cine phase contrast (PC) MR imaging [36] was performed to quantify chamber blood velocity. Published studies support to a large extent the validation of these techniques against ground truth [37,38], and the application of these techniques in other animal models (rodent [39,40], canine [41], porcine [42]) and in the clinic [43,44]. In this study, optimized imaging protocols and analysis methodology based on these established techniques were designed to provide a standardized, and robust assessment of regional cardiac function in naïve NHPs. A set of imaging biomarkers that are clinically relevant for HF research was defined and statistical analyses (intra-subject test-retest repeatability and inter-subject variability) were conducted on these biomarkers. The biomarkers were selected to broadly characterize regional systolic pump behavior (peak circumferential and longitudinal shortening, average systolic strain-rate, peak torsion, circumferential and longitudinal strain dyssynchrony) as well as regional diastolic relaxation and filling behavior (average diastolic strain-rate, diastolic peak mitral inflow velocity (E)/circumferential strain-rate (CSR) ratio, diastolic E/ longitudinal strain-rate (LSR) ratio, peak untwist rate, time-to-peak untwist rate, deceleration time), thus providing comprehensive insight into LV function in naïve NHPs. Finally, based on the statistical analyses, a recommendation on a subset of robust functional biomarkers that can be used for evaluating novel therapeutics is also presented.

## Materials and Methods

### Ethics Statement

The following experiments were conducted in accordance with the Institutional Animal Care and Use Committee at Merck & Co and Maccine Pte. Ltd. The current study, in addition to its protocol, was approved by the Institutional Animal Care and Use Committee at Merck & Co (Permit Number: 12102130610018) and the testing facility at Maccine (Maccine Pte Ltd, Singapore; IACUC number: 281–2012). No animals were sacrificed for the purpose of this experiment. Trained veterinarians and animal technicians were involved in the care of the animals as well as all the animal procedures performed during this study.

### Animal

Eight naïve Vietnamese cynomolgus macaques (weight: 3.6 ± 0.2 kg (mean ± standard deviation (SD)), age: 5.5 ± 0.5 years (mean ± SD), sex: female, resting heart rate: 131 ± 17 beats per minute (bpm) (mean ± SD)) were imaged. All animals selected in this study were naïve, close in age, and had no history of illnesses, pharmaceutical interventions, or fluctuating body weights. Animals were housed in temperature- (18°-26° C) and humidity-controlled (30–70%) rooms that maintained on a 12:12 light/dark cycle with lights on at 7:00AM at Maccine facility. Mirrors were placed outside the home cage, and cage toys/manipulative enrichment, such as Kong Toys, Flexi keys, were provided and rotated every week. Radios and televisions were also used as supplementary enrichments. Animals were fed with a diet of monkey chow free of animal protein, as well as a controlled amount of fruits or vegetables, offered twice daily. Aside from daily fruit rations, frozen homemade treats (i.e. fruits, raisins, cereals, etc.) were provided once a week. Mains tap water was offered ad libitum. Animals were anesthetized using ketamine HCl (10 mg/kg IM, induction), and isoflurane (2% in medical air, maintenance) during imaging. The animals were ventilated by a SurgiVet V727000 MRI 2550 ventilator and bellows system (Surgivet, Dublin, OH). They were kept warm by using a warm air blower, and their heart rate, respiratory rate, blood oxygenation level, and end-tidal CO2 level were monitored every minute during the entire imaging session using an MR imaging compatible GE Datex-Ohmeda physiological monitoring system (GE Healthcare, WI).

### Experimental Design

All imaging experiments were performed using the 3 T Trio MR imaging scanner (Siemens Medical Solutions, Erlangen, Germany) equipped with gradient strengths of up to 45 mT/m and maximum slew rates of 200 mT/m/s. The spine matrix coil and the 4-channel phased array body flex matrix coil were used for imaging. The animals were placed in the supine position on the MR scanner table. The imaging protocol was executed twice (i.e. test and retest scans) in each animal. The test and retest scans were conducted sequentially in the same imaging session. Following the test scan, the animal was pulled out of the scanner, the coils were repositioned and the animal was re-landmarked to the magnet isocenter prior to the retest scan. All animals were fasted around twelve hours prior to the start of the test scan.

The following regional functional biomarkers were quantified: 1) peak strain, 2) peak torsion, 3) average systolic CSR and diastolic CSR, 4) average systolic LSR and diastolic LSR, 5) strain dyssynchrony, 6) E/CSR and E/LSR ratios, 7) peak untwist rate, 8) time-to-peak untwist rate, and 9) deceleration time. Test-retest repeatability and inter-subject variability analyses were carried out on each of these biomarkers to evaluate their performance.

### MRI

Localizers along three short axis (SA) slices (at the apex, mid and base), and three long axis (LA) slices (2-chamber, 4-chamber, and LA slice with the LV outflow tract (LVOT) in view) were prescribed. The SA slices were separated by 4 mm, and the center of the middle slice was positioned to be at the center of the LV LA.

Cine (multi-phase, i.e multiple images acquired over the cardiac cycle) structural images of the final selected three SA and the 2-chamber and 4-chamber LA slices were also acquired. These acquisitions were conducted during breath-holds to eliminate breathing-related motion artifacts. The breath-holds were enforced by taking the animal temporarily off the ventilator. A multi-phase, gated, segmented gradient-echo pulse sequence was used. The typical imaging parameters used were: FOV: 150 mm × 150 mm, imaging matrix: 208 × 208, slice thickness: 4 mm, TR/TE: 7.3 ms/3.39 ms, flip angle: 12°, bandwidth: ±46.2 KHz, temporal resolution: 36.3 ms, segments: 5, phases: 12, averages: 2. No parallel imaging was used. All cine data was acquired during ventilator-induced breath-holds, which were on average around 40 s long.

A gated, multi-phase, segmented gradient echo pulse sequence with a 1-2-1 SPAMM tagging [32,33] preparation sequence was employed to image myocardial displacement. Two sets of tagging datasets with orthogonal in-plane tagging modulations were acquired for each of the three SA slices and for the 2-chamber and 4-chamber LA slices. The imaging parameters were selected to achieve good spatial resolution and temporal resolution while maintaining acceptable signal-to-noise ratio (SNR) and scan times. The selected imaging parameters were: FOV: 150 mm × 150 mm, imaging matrix: 208 × 208, slice thickness: 4 mm, TR/TE: 7.26 ms/3.39 ms, flip angle: 12°, bandwidth: ±45 KHz, temporal resolution: 21.78 ms, segments: 3, phases: 19, tag separation: 4 mm. No parallel imaging was used. All tagging data was acquired during ventilator-induced breath-holds, which were on average around 35 s long.

A gated, multi-phase, PC MR imaging pulse sequence was employed to image LV chamber velocity [36]. Two PC MR imaging datasets with velocity sensitivity along orthogonal in-plane directions were acquired for each of the three prescribed LA slices. In addition, for each slice, the acquisition of the two above mentioned datasets were repeated, but this time with a fixed trigger delay. This temporal interleaving was used to increase the apparent temporal resolution of the PC datasets. The imaging parameters used were identical to the tagging sequence with the exception of: imaging matrix: 128 × 128, TR/TE: 14.25 ms/4.49 ms, flip angle: 15°, temporal resolution: 14.25 ms, segments: 1, phases: 30, velocity encoding gradient parameter (VENC): 100 cm/s, and trigger delay for interleaved scans: 7 ms,. No parallel imaging was used. Ventilator breath-hold was induced during the middle section of the scan, lasting on average around 40 s.

### Image Analysis

#### LV Global Structure and Function

The Cine structural imaging datasets for all animals were analyzed using the freely available software Segment v1.9 R3612 (http://segment.heiberg.se) [45] to quantify LV length, diameter, wall thickness (averaged over all three SA slices), end-diastolic volume (EDV), end-systolic volume (ESV), stroke volume (SV), ejection fraction (EF), cardiac output (CO), and LV mass. The analysis was repeated for both test and retest datasets.

#### LV Displacement

The tagged imaging datasets were analyzed using HARP methodology [34,35] to quantify myocardial displacement. For HARP analysis, one of the harmonic peaks obtained from the Fourier transform of the tagged images was isolated using a flat-topped circular band-pass filter of radius 22 pixels with Gaussian boundaries of width 3 pixels (total filter bandwidth = 50 pixels). The isolated peaks were zero-padded to image size 256 × 256 and reconstructed to obtain a series of (complex-valued) harmonic images. This filter size affords a dynamic strain measurement of around ±53%, which is well within the expected strain range of normal as well as diseased NHPs [46]. As a result, the selected filter adequately samples the tagging modulation function, and the interpolation function used (due to zero-padding) provides faithful reconstruction of the HARP signal, within limits of the strain resolution. The filter size used provides an underlying in-plane strain resolution of the order of 3.4 mm × 3.4 mm. For each slice, the two orthogonal tagging datasets were analyzed to produce a two-dimensional (2-D) HARP value for each material point in the image.

For each SA slice, a user-defined mesh with three layers (endocardium, mid-wall, and epicardium) and four (apical) to six (mid and basal) segments was superimposed on the myocardium at the first time frame. The mesh was defined by 24 (apical) to 36 (mid and basal) material points. The 2-D displacement path lines of the mesh material points were then obtained by tracking their 2-D HARP values [47] in time using a neighborhood search approach. The tracked layers are super-imposed on grid tag lines that are synthetically generated from the harmonic phase images [48]. For each LA slice, the 2-D displacement path lines of all voxels in the myocardium were tracked.

#### LV Torsion

For apical and basal SA slices, the center of rotation at any time point was defined as the center of mass of the mesh material points at that time point for the given slice. The rotation of each mesh material point was then calculated [49,50]. Positive rotation corresponded to clockwise rotation, while negative rotation corresponded to anti-clockwise rotation. The average LV rotation of all mesh material points lying on that slice was then computed. Finally, the average LV torsion, defined as the circumferential-longitudinal shear angle in degrees, was computed [50]. Torsion curves from base to apex (Φ_base-apex_), mid to apex (Φ_mid-apex_), and base to mid (Φ_base-mid_) were computed. Peak torsion from each of these temporal curves was then quantified. The derivative of the base-apex torsion curve with respect to time was computed and the peak untwist rate during early diastole was quantified. The time-to-peak untwist rate was also quantified.

#### LV Strain, Strain-Rate and Strain Dyssynchrony

Average regional circumferential strain was computed from the SA tagged slices, and regional longitudinal strain was computed from the LA slices. Regions defined on the SA and LA slices were in accordance to the American Society of Echocardiography standardized cardiac segmentation guidelines. The strain was computed in the Lagrangian sense using the formula [47]:

$$\varepsilon \left(p_{\text{i}},p_{\text{j}},{\text{t}}_{\text{n}}\right)=\frac{f\left(p_{\text{i}}\left({\text{t}}_{\text{n}}\right),p_{\text{j}}\left({\text{t}}_{\text{n}}\right)\right)}{f\left(p_{\text{i}}\left({\text{t}}_{0}\right),p_{\text{j}}\left({\text{t}}_{0}\right)\right)},$$ (1)

where *f*(.) is the functional that represents the circumferential distance between two position vectors **p**_i_ and **p**_j_ for SA slices and the longitudinal distance between two position vectors **p**_i_ and **p**_j_ for LA slices. For the SA slices, the position vectors **p**_i_ and **p**_j_ were selected such that they are circumferentially adjacent points on the user-defined mesh for each layer. Thus for each defined region, the average strain from six combinations of points (two combinations per layer) was computed. For the LA slices, voxel-wise strain was computed. For each voxel position vector **p**_i_, eight neighborhood position vectors **p**_j_ were selected and the average longitudinal strain from the eight combinations of points was computed. Finally, the average longitudinal strain within each defined segment was computed as the average strain of all pixels within that segment. Dense pixel-wise Eulerian strain was also computed for the SA slices for visual inspection of data quality. This was obtained by computing gradients of the harmonic phase vectors [35]. Regional strain-rates were obtained by computing the derivative in regional Lagrangian strain with respect to time. The strain-rates were averaged prior to peak strain (systolic strain-rate) and post peak strain (diastolic strain-rate). In addition, the standard deviation in time-to-peak strain (circumferential and longitudinal) in the defined axial segments for basal, mid and apical levels was computed as a measure of strain dyssynchrony for that level.

#### LV Chamber Hemodynamics

The PC images were used to generate blood velocity maps in the LV chamber. The velocity component along the direction of the aortic outflow tract and the mitral inflow tract was computed from the in-plane velocity measurements for the three LA slices. Velocity-time curves were then generated for aortic outflow and mitral inflow. For aortic outflow, the computed outflow velocity was averaged within a user-defined region of interest in the outflow tract from the LVOT LA slice. For mitral inflow, the computed inflow velocity was averaged within two user-defined regions of interest defined near the mitral valve on the 2-chamber and the 4-chamber LA slices. Composite velocity-time curves were then generated by piecing together the aortic velocity-time curve during systole until the aortic flow velocity returns to zero, and the mitral velocity-time curve after that point during the remainder of the cardiac cycle. Deceleration time was then computed as the time from peak mitral inflow velocity to the time the mitral velocity curve reaches zero.

#### Functional Strain-Torsion-Velocity Relationships

To characterize cardiac function in the imaged cohort, functional inter-relationships between global average circumferential strain, global average torsion, and composite velocity was studied. All rotation-time, strain-time and velocity-time curves were temporally smoothed using a higher-order polynomial fitting function, and temporally scaled using a constant factor to a reference mean heart-rate of 134 bpm to account for differences in heart-rates between scans and between animals. Temporal relationships were investigated by simultaneously plotting the time curves for all three quantities. The relative rate change for rotational mechanics and strain mechanics during systole and diastole were studied by plotting torsion versus circumferential strain to generate a partial loop. The relative amplitudes and rate relationships between mechanics and hemodynamics was also studied by generating partial loops of torsion versus composite velocity, and circumferential strain versus composite velocity.

#### Statistical Analysis

For the quantified global structural biomarkers from the cine imaging datasets, a standard two-tailed t-test statistical analysis was performed to detect any significant differences between test and retest values at the p<0.05 significance level. Intra-subject repeatability and inter-subject variability statistics were next performed on all functional biomarkers defined above. The intra-subject repeatability statistics were performed on the test-retest datasets. The following methods were employed:

1. Bland-Altman analysis. The mean (X+Y)/2 versus the difference X − Y of the test and retest data for each biomarker was plotted, and the average of the difference and the standard deviation (SD) of the difference were computed and the average ± 1.96 SD lines were plotted. The linear regression trend-line was also plotted. For peak strain, strain-rate and E/strain-rate ratios, all measurements obtained from each segment and each slice were used as independent data points in the Bland-Altman plot. For peak torsion, peak untwist rate and time-to-peak untwist rate, three regional measurements were plotted for each animal (base-apex, mid-apex and base-mid). For strain dyssynchrony, one measurement for each of the three slice levels was plotted per animal. For deceleration time, one measurement per animal was computed from the mitral inflow velocity curve and plotted.
2. Intra-subject concordance correlation coefficient (CCC) analysis. For intra-subject CCC analysis, the strain biomarkers were averaged regionally to obtain one measurement for apical, mid and basal slices and the CCC was computed for these averaged values [51].

The inter-subject variability was assessed by computing the mean and SD of each biomarker within the imaged NHP cohort (only the test data was used here) and the relative standard deviation.

Prospective statistical calculations to evaluate the minimum sample size for a hypothetical population (undergoing a treatment, for example) that would be needed to detect a 15% change in a biomarker with 80% statistical power (β = 0.2) and statistical significance of P<0.05 (α = 0.05) using a two-tailed t-test were also performed. The standard deviation in the measurement of the imaging biomarker was also assumed to be identical for both groups (s1 and s2). The effect size (d) was calculated using the following formula:

$$\text{d}=\frac{0.15\times {\bar{\text{x}}}_{1}}{{\text{s}}_{1}}\;,$$ (2)

where $\bar{{\text{x}}_{1}}$ is the mean of the biomarker for the imaged NHP cohort in this study. The sample size (n_2_) was then computed (using a one-sided hypothesis test) as follows:

$${\text{n}}_{2}\geq 2\times \frac{{\left({\text{z}}_{1-\propto }-{\text{z}}_{\beta }\right)}^{2}}{{\text{d}}^{2}},$$ (3)

where z is the standard score at a given level (in this case 1-α and β).

## Results

### Physiological Data and Global Cardiac Parameters

**Fig 1:**
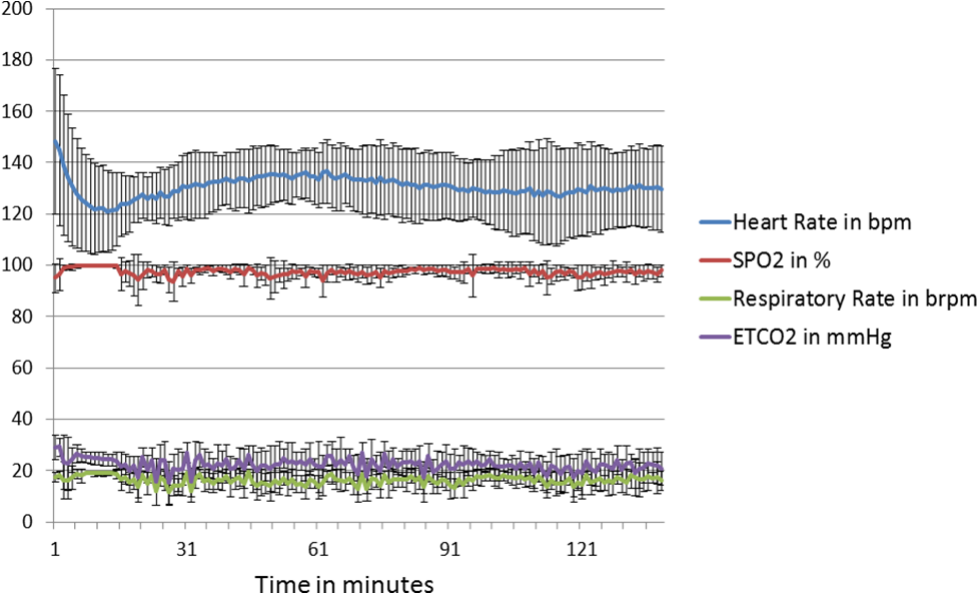
Temporal graphs depicting stability of physiological parameters during the course of the imaging study. Physiological data (heart rate, respiratory rate, oxygen saturation (SP0_2_), and CO_2_ levels) from the eight NHPs recorded during the MR imaging experiments. Solid line depicts the mean data, while the error bars denote the ± one standard deviation. Measurement sampling was obtained every minute during the study.

The physiological data recorded during all imaging sessions is illustrated in Fig 1. The plots demonstrate good consistency in measured physiological parameters for the imaged NHP cohort, while excellent stability in these readouts is also achieved throughout the length of the imaging session, indicating low fluctuations in cardiac hemodynamics during the length of the experiment. The measured heart-rate, body-weight and quantified cardiac global structural and functional parameters from standard cine MR imaging datasets are shown in Table 1. No significant differences in the quantified cardiac parameters between test and retest datasets were observed confirming reproducible anesthetic conditions and its effect on cardiac behavior. Relative standard deviation of ejection fraction within the imaged cohort were low (10% for test datasets, and 13% for retest datasets) confirming close similarity in global cardiac functional behavior within the imaged naïve cohort.

**Table 1:** Body weights, heart rates, LV structural characteristics, ejection fraction, cardiac output and LV mass from imaged NHPs.

Vital, structural and functional parameters in imaged NHPs (n = 8) (mean±SD)		
	Test	Retest
**Body weight (kg)**	3.58±0.23	3.65±0.27
**Heart rate (bpm)**	149±17	150±18
**Length (ED/ES) (mm)**	33.59±2.34/29.33±2.07	33.74±1.21/29.6±1.45
**Diameter (ED/ES) (mm)**	25.67±1.79/23.13±2.84	22.30±1.66/22.53±1.79
**Wall thickness (ED/ES) (mm)**	4.09±0.49/6.56±0.85	3.97±0.45/6.46±1.06
**EDV (ml)**	4.75±1.16	4.75±1.04
**ESV (ml)**	2±0.75	1.88±0.83
**SV (ml)**	3±1.07	2.75±0.46
**EF (%)**	62±9.44	59.5±11.95
**CO (l/min)**	0.43±0.1	0.43±0.12
**LV mass (g)**	7.5±1.2	7.62±0.92
**Peak filling rate (ml/s)**	28.12±7.41	27.62±9.10
**Peak ejection rate (ml/s)**	29.12±7.43	27.5±7.25

### Cardiac Strain

**Fig 2:**
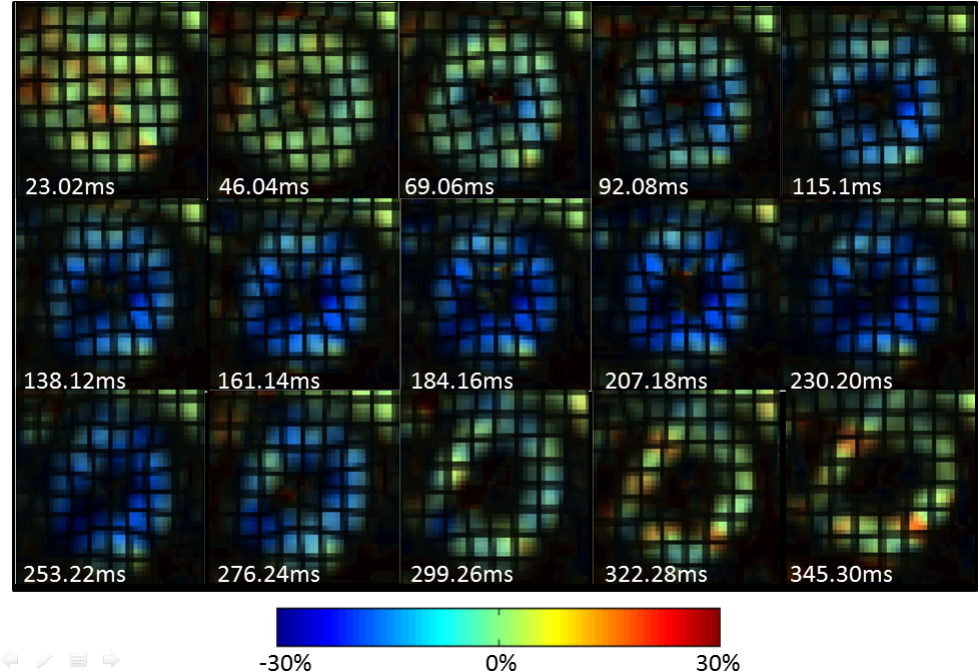
Circumferential strain evolution over a cardiac cycle in a representative NHP. Circumferential strain color maps with tag overlay in a mid-ventricular short-axis slice of a representative NHP over the entire cardiac cycle depicting systolic compression and diastolic relaxation.

Successful optimization of the MR pulse sequences was achieved to realize functional quantification of myocardial motion and LV chamber blood velocity in NHP hearts. A sequence of Eulerian circumferential strain color maps in a mid-ventricular short axis slice with synthetic grid tag overlay in one representative animal is depicted in Fig 2, where good tag contrast throughout the cardiac cycle with minimal tag decay is evident. The synthetic tags also confirm the good quality of the underlying HARP data (derived from the tagged images) throughout the cardiac cycle, including end-diastole. The data also confirm that the tag spacing selected was optimal to achieve acceptable strain resolution, while maintaining insensitivity to through-plane tilt motion for the thin SA slices that were prescribed in the NHP hearts. The strain results highlight good signal-to-noise ratio and good HARP filter design with no significant Gibbs ringing artifacts.

**Fig 3:**
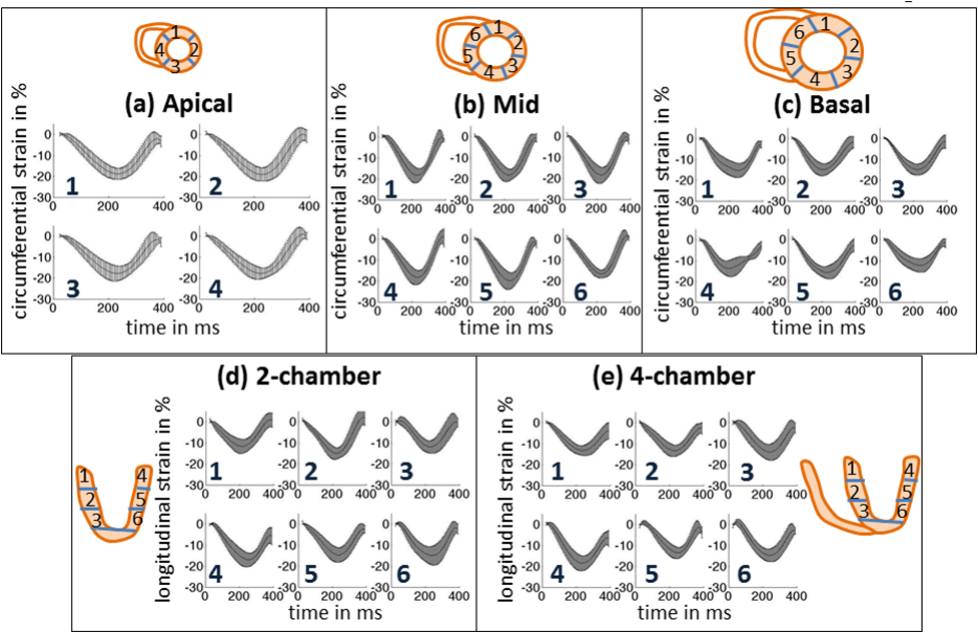
Temporal average strain evolution curves. Strain curves in defined regions on short-axis (circumferential) and long-axis (longitudinal) slices (per American Society of Echocardiography guidelines) for the imaged NHP cohort (n = 8). The curves are temporally scaled to a standard heart rate. The mean curve is then depicted as a solid line and the error bars represent one standard deviation measurement. The sampling rate for the error bars is set arbitrarily.

**Fig 4:**
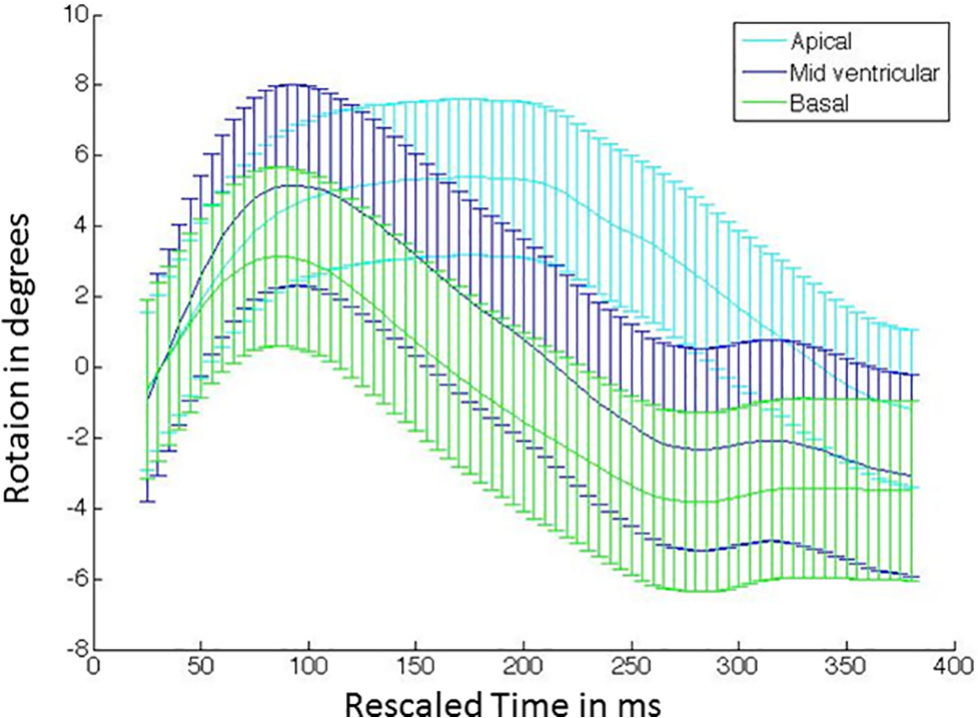
Temporal average rotation curves. The curves are depicted for representative short axis slices located at the LV base, mid and apex for the imaged NHP cohort (n = 8). The curves represent the mean value, while the error bars represent one standard deviation measurement. The sampling rate for the error bars is set arbitrarily.

Regional strain-time curves in defined segments for the three SA slices (circumferential strain) and the two LA slices (longitudinal strain) are shown in Fig 3, and regional rotation-time curves for three SA slices are shown in Fig 4. In Fig 3, the solid lines represent the average value for all 8 NHPs, while the error bars depict the ± one standard deviation values. Circumferential shortening occurs during systole followed by circumferential expansion during diastole. This trend is observed in all segments and all slices. Peak circumferential shortening occurs between 45–65% of the cardiac cycle. Regional differences in peak circumferential strain between segments within each SA slice were minimal and not statistically significant. Similar to the circumferential strain behavior, longitudinal shortening occurs during systole followed by longitudinal expansion during diastole. This trend is observed in all segments for both LA slices. Peak longitudinal shortening also occurs between 45–65% of the cardiac cycle. Peak longitudinal strain between anterior, posterior, lateral and septal regions was minimal and not statistically significant. As seen in Fig 4, all three slices exhibit early systolic clockwise rotation. The apex achieves maximum peak rotation, while the base achieves minimum peak rotation. The mid-base regions begin untwisting early and continue to rotate in the anti-clockwise direction beyond the original zero rotation point, thus achieving a maximum peak negative rotation during mid-diastole. Similar behavior is observed in humans [52].

### Cardiac Hemodynamics

**Fig 5:**
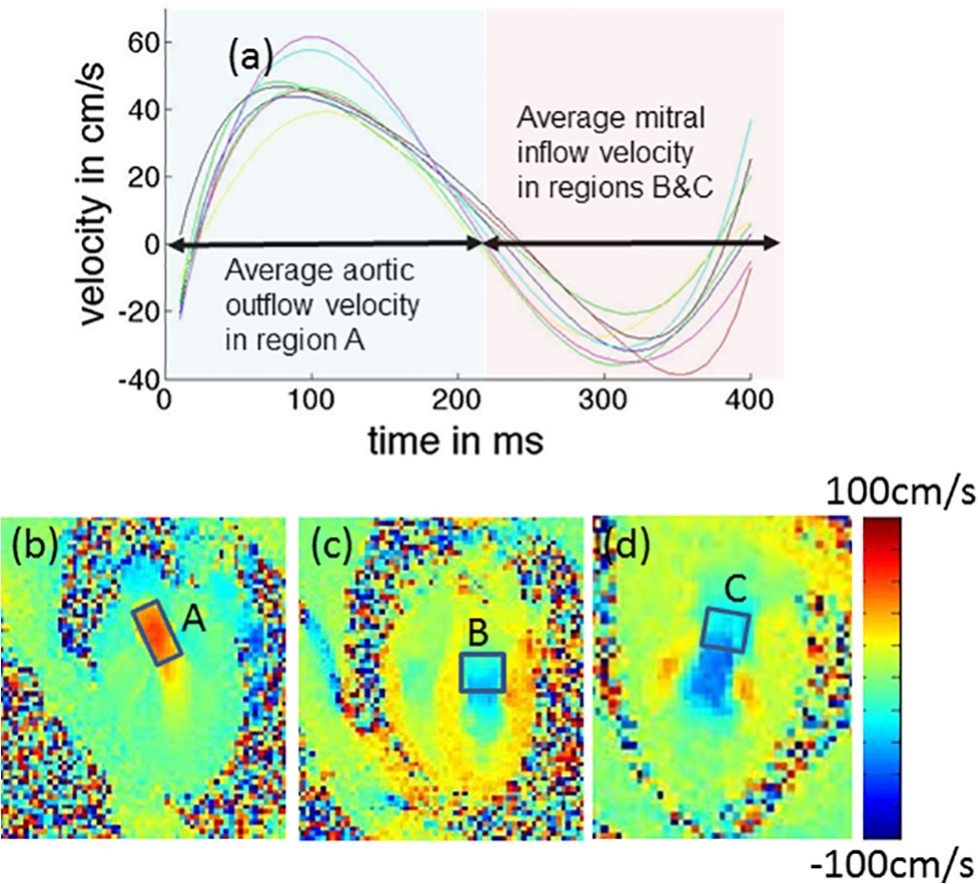
Temporal flow velocity curve depicting aortic outflow and mitral inflow hemodynamics. Composite flow velocity curve is shown in (a). The curve is temporally scaled to a standard heart rate. Solid line depicts mean value for all 8 NHPs, while error bars depict the ± one standard deviation values. The first section of the curve is obtained by averaging the velocity along the direction of the LV outflow tract over the region of interest (ROI) defined as shown in (b), while the second section of the curve is obtained by averaging the velocity along the direction of the long axis over the ROIs defined in (c) and (d).

Composite velocity-time curves are plotted in Fig 5. Peak aortic outflow occurs during early-systole at around 15–25% of the cardiac cycle and peak mitral inflow occurs during early diastole at around 67–80% of the cardiac cycle. The temporal trend in velocity was similar across all animals with a relatively low spread in peak outflow and inflow velocities. The aortic outflow velocities were found to be higher than the mitral inflow velocities.

### Inter-relationships between Myocardial Mechanics and Chamber Hemodynamics

**Fig 6:**
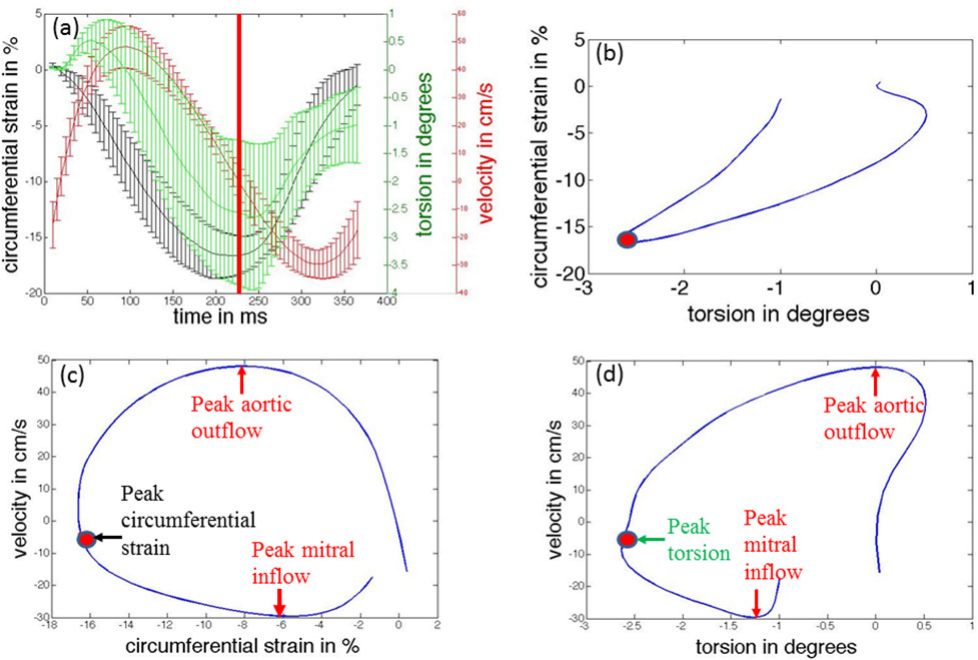
Temporal inter-relationships between LV mechanics and LV chamber hemodynamics. (a) Comparisons of temporal responses of circumferential strain, torsion and composite velocity (temporally scaled to a standard heart rate). Solid line depicts the mean value, and error bars depict the ± one standard deviation values. The red vertical solid line corresponds to the time of peak circumferential strain (end-systole). A partial loop depicts (b) mean torsion versus mean circumferential strain, (c) mean circumferential strain versus mean velocity, and (d) mean torsion versus mean velocity. The red dots in (b-d) correspond to the red line in (a). Key timing events corresponding to peak aortic outflow, peak mitral inflow, peak circumferential strain and peak torsion are also indicated in the loops (c-d).

Functional relationships between average base-to apex torsion, average circumferential strain (averaged over all slices and segments), and composite velocity are highlighted in Fig 6. In Fig 6A, the temporal curves of the mean of these three quantities for all eight animals are displayed by solid lines, and error bars indicate the standard deviation in the quantity measured at each resampled time point within the entire cohort. The curves illustrate the temporal inter-relationships between LV mechanics (rotational and circumferential), and LV hemodynamics. We observe that once systolic compression and torsion begins, peak aortic outflow velocity is quickly reached early on in systole at around 22% of the cardiac cycle. The heart then continues to reduce its dimensions and to twist, thus actively pushing more blood out of the LV and into the aorta. Peak circumferential shortening and peak torsion are observed at around 46% and 52% of the cardiac cycle respectively. Mitral inflow then begins at around 50% of the cardiac cycle. The untwisting motion in the heart during early diastole coincides with rapid filling of blood into the LV and is associated with increased LV circumferential strain. Peak mitral inflow velocity is observed at around 70% of the cardiac cycle.

The mean torsion versus the mean circumferential strain is plotted in Fig 6B. During early systole, a non-linear relationship between the two quantities exists, with greater change in circumferential strain observed for smaller change in torsion. This behavior continues until peak aortic flow velocity is achieved. After this time point, circumferential strain and torsion exhibit a linear relationship during the remainder of systole. In addition, we observe that the rate of change in both quantities, normalized to their respective peak systolic values remain constant, i.e. if the circmferential strain changes by 1% of the peak systolic strain, the torsion also changes by 1% of the peak torsion. During early-mid diastole, a linear relationship is maintained but with a slower torsion rate (i.e. if the circumferential strain changes by 1% of peak systolic strain, the torsion changes by 0.75% of peak torsion.

The mean circumferential strain versus the mean velocity is plotted in Fig 6C, while the mean torsion versus the mean velocity is plotted in Fig 6D. These figures highlight the relative behavior between LV mechanics and hemodynamics. It is observed that peak aortic outflow velocity is reached when approximately 45% of the peak circumferential shortening and 25% of torsion has occurred. On average for all animals, peak aortic outflow velocity occurs around 45% into systole; thus the rate of change in mean circumferential shortening remains relatively the same throughout systole. However, the rate of change in torsion is much higher during the second half of systole. During diastole, peak mitral inflow velocity is reached when 70% circumferential expansion and 50% of untwisting has been completed. On average for all animals, peak mitral inflow velocity occurs around 45% into diastole; thus the rate of circumferential expansion is much higher during the isovolumic relaxation and acceleration phase of rapid filling. However, nearly 50% of untwisting takes place post peak mitral inflow.

### Biomarker Regional Variability

**Fig 7:**
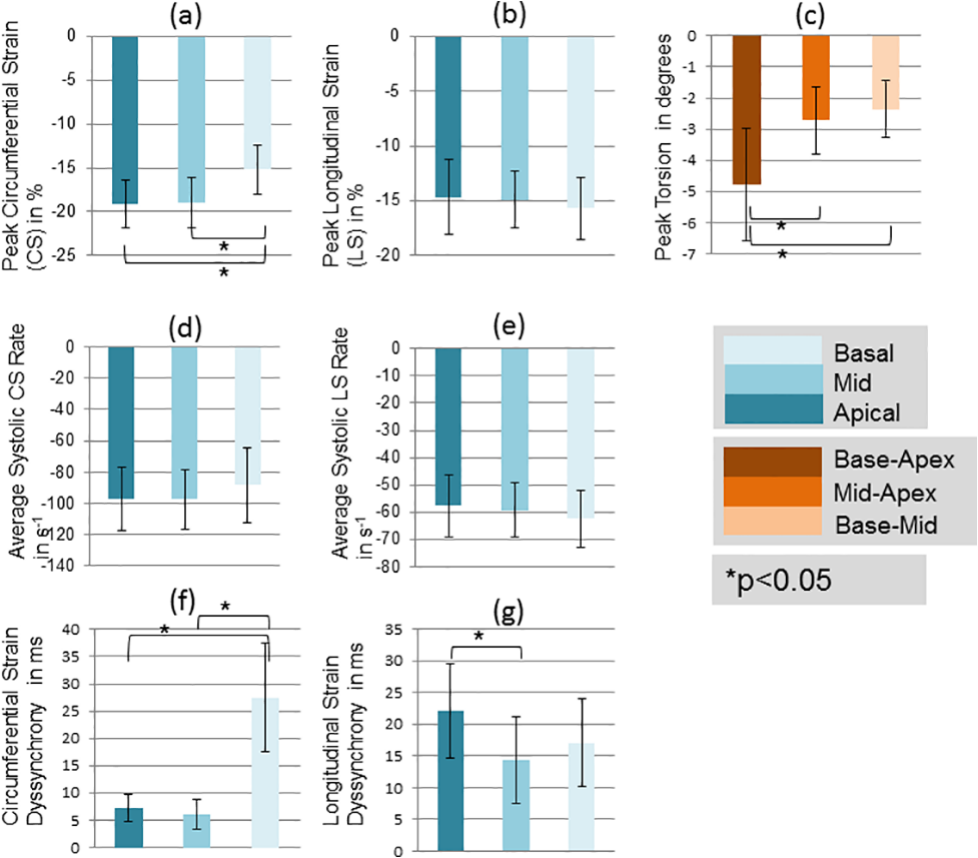
Spatial heterogeneity of quantified systolic biomarkers. Comparisons of quantified systolic biomarkers averaged over three regions: apical, mid-ventricular and basal. Asterisk denotes a significant difference in values at the p<0.05 significance level using a 2-tailed paired t-test. Regional differences are observed for peak circumferential strain, peak torsion, and strain dyssynchrony.

**Fig 8:**
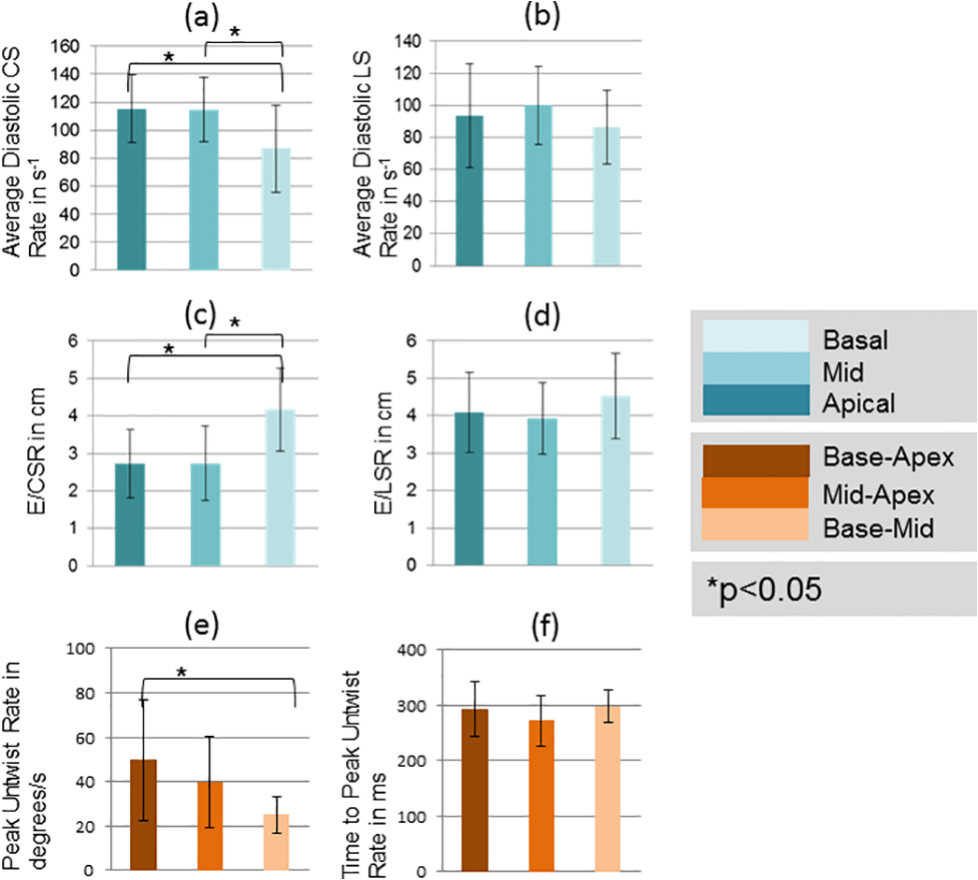
Spatial heterogeneity of quantified diastolic biomarkers. Comparisons of quantified diastolic biomarkers averaged over three slices: apical, mid-ventricular and basal. Asterisk denotes a significant difference in values at the p<0.05 significance level using a 2-tailed paired t-test. Regional differences are observed for average CSR, and peak untwist rate.

Results of regional variability in the quantified systolic and diastolic biomarkers outlined in the Methods section are shown in Figs 7 and 8 respectively. Deceleration time was not plotted as only one value was obtained per animal from the mitral inflow velocity curve and there were no regional values calculated. The calculated mean±SD for deceleration time was 65±12 ms. Among the systolic biomarkers, the basal circumferential strain was found to be significantly lower (P< 0.05; 2-tailed t-test) while circumferential dyssynchrony was significantly higher compared to the apical and mid-ventricular regions (P< 0.05; 2-tailed t-test). Longitudinal strain was the highest at the base, although its value was not statistically different to other regions. Further, longitudinal dyssynchrony was lowest in the mid-ventricular region, but it was only statistically different from the apical and not the basal (P< 0.05; 2-tailed t-test). No significant differences in systolic strain-rates were observed. Peak base-apex torsion was significantly higher than mid-apex and base-mid torsion as expected (P < 0.05; 2-tailed t-test).

Among the diastolic biomarkers, diastolic CSR in the base was significantly lower as compared to other regions (P < 0.05; 2-tailed t-test), while E/CSR was significantly higher in the base (P < 0.05; 2-tailed t-test). No significant regional differences were observed in diastolic LSR and E/LSR. Peak untwist rate from base-apex was the highest while base-mid was the lowest and were statistically different (P < 0.05; 2-tailed t-test). No regional differences were observed in time-to-peak untwist rate.

The regional computed values of all above discussed biomarkers are also tabulated for each imaged animal in **Tables A-N** in **S1 File**.

### Biomarker Performance

**Fig 9:**
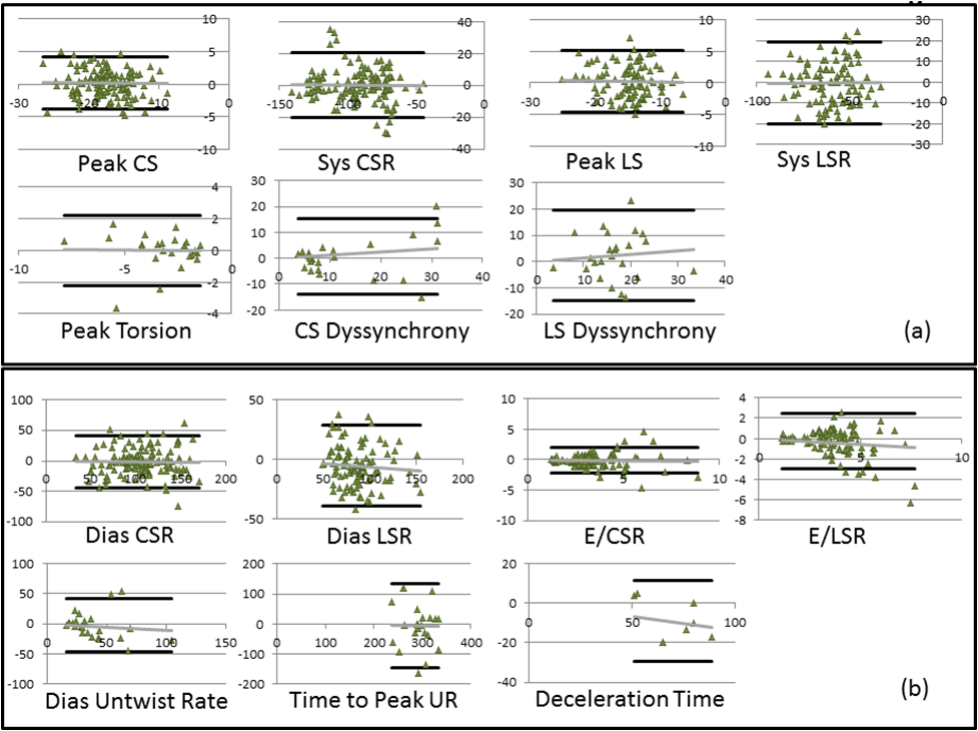
Plots depicting Bland-Altman repeatability analysis for all quantified biomarkers. Bland-Altman plots of quantified (a) systolic biomarkers, and (b) diastolic biomarkers. The solid gray line represents the linear regression trend line that associates the difference between the two measurements with the average between the two measurements. The solid black lines represent the standard deviations of the difference between the two measurements. Sys = systolic, Dia = diastolic, CS = circumferential strain, LS = longitudinal strain, CSR = circumferential strain-rate, LSR = longitudinal strain-rate.

Results obtained from intra-subject repeatability analyses between test and retest scans are shown in Tables 2 and 3 for the systolic and diastolic biomarkers respectively, while results of the inter-subject variability analyses within the imaged NHP cohort are shown in Tables 4 and 5 for the systolic and diastolic biomarkers respectively. The Bland Altman plots for all biomarkers are shown in Fig 9.

**Table 2:** Results from intra-subject repeatability analyses for systolic biomarkers.

Intra-Subject Repeatability Statistics (Systolic)				
	**Peak CS (%)**	**Systolic CS Rate (%/s)**	**Peak LS (%)**	**Systolic LS Rate (%/s)**
**Bland Altman (mean diff/1.96SD)**	0.13/4.02	0.11/20.51	0.16/4.93	-0.64/18.52
Bland Altman [limit of repeatability (1.96*SD)/mean value of parameter]	-0.23	-0.22	-0.34	-0.33
**CCC_apical/[lbound,ubound]**	0.75/[0.3,0.9]	0.97/[0.86,0.99]	0.88/[0.6,0.97]	0.7/[0.13,0.92]
**CCC_mid/[lbound,ubound]**	0.89/[0.6,0.97]	0.97/[0.87,0.99]	0.73/[0.2,0.93]	0.83/[0.6,0.97]
**CCC_basal/[lbound,ubound]**	0.87/[0.5,0.97]	0.89/[0.58,0.97]	0.9/[0.6,0.98]	0.89/[0.6,0.97]
	**Peak Torsion (degrees)**	**CS Dyssynchrony (ms)**	**LS Dyssynchrony (ms)**	
**Bland Altman (mean diff/1.96SD)**	0/2.22	0.76/14.39	2.39/17.31	
Bland Altman [limit of repeatability (1.96*SD)/mean value of parameter]	-0.68	1.09	1.04	
**CCC_apical/[lbound,ubound] (base-apex for peak torsion)**	0.62/[-0.07,0.91]	0.28/[-0.46,0.79]	0.34/[-0.37, 0.8]	
**CCC_mid/[lbound,ubound] (mid-apex for peak torsion)**	0.45/[-0.3,0.86]	0.21/[-0.47,0.74]	0.1/[-0.58, 0.71]	
**CCC_basal/[lbound,ubound] (base-mid for peak torsion)**	0.83/[0.37,0.96]	-0.12/[-0.66,0.49]	-0.13/[-0.7,0.54]	

**Table 3:** Results from intra-subject repeatability analyses for diastolic biomarkers.

Intra-Subject Repeatability Statistics (Diastolic)				
** **	**Diastolic CS Rate (%/s)**	**Diastolic LS Rate (%/s)**	**E/CSR (m)**	**E/LSR (m)**
**Bland Altman (mean diff/1.96SD)**	-1.64/42.37	-3.11/46.28	-0.12/2.13	-0.28/2.7
Bland Altman [limit of repeatability (1.96*SD)/mean value of parameter]	0.41	0.39	0.64	0.72
**CCC_apical/[lbound,ubound]**	0.92/[0.7,0.98]	0.84/[0.46,0.96]	0.64/[0.39.0.81]	0.57/[0.32,0.74]
**CCC_mid/[lbound,ubound]**	0.86/[0.51,0.97]	0.83/[0.41,0.96]	0.51/[0.27,0.68]	0.74/[0.54,0.86]
**CCC_basal/[lbound,ubound]**	0.87/[0.51,0.97]	0.84/[0.44,0.96]	0.65/[0.45,0.78]	0.48/[0.19,0.69]
** **	**Peak Untwist Rate (degrees/s)**	**Time-to-Peak Untwist Rate (ms)**	**Deceleration Time (ms)**	
**Bland Altman (mean diff/1.96SD)**	-2.9/44.41	5.2/140.55	-8.8/20.5	
Bland Altman [limit of repeatability (1.96*SD)/mean value of parameter]	1.12	0.48	0.29	
**CCC/[lbound,ubound]**	0.55/[0.21,0.77]	-0.25/[-0.58,0.15]	0.61/[0.1,0.87]	

**Table 4:** Results from inter-subject variability analyses for systolic biomarkers.

Inter-Subject Variability Statistics (Systolic)				
	**Peak CS (%)**	**Systolic CSR (%/s)**	**Peak LS (%)**	**Systolic LSR (%/s)**
**Apical (RSD/minimum sample size)**	14/8	20/17	24/22	20/16
**Mid (RSD/minimum sample size)**	15/9	19/16	16/12	17/12
**Basal (RSD/minimum sample size)**	18/14	26/20	18/13	16/12
** **	**Peak Torsion (degrees)**	**Circumferential Strain Dyssynchrony (ms)**	**Longitudinal Strain Dyssynchrony (ms)**	
**Apical (RSD/minimum sample size) (base-apex for peak torsion)**	37/15	33/12	33/12	
**Mid (RSD/minimum sample size) (mid-apex for peak torsion)**	39/17	44/22	47/24	
**Basal (RSD/minimum sample size) (base-mid for peak torsion)**	39/16	35/14	40/17	

**Table 5:** Results from inter-subject variability analyses for diastolic biomarkers.

Inter-Subject Variability Statistics (Diastolic)				
	**Diastolic CSR (%/s)**	**Diastolic LSR (%/s)**	**E/CSR (m)**	**E/LSR (m)**
**Apical** (**RSD/minimum sample size)**	20/18	34/48	25/7	35/14
**Mid** (**RSD/minimum sample size)**	20/16	24/24	27/8	35/14
**Basal** (**RSD/minimum sample size)**	35/50	26/28	43/21	36/15
** **	**Peak Untwist Rate (degrees/s)**	**Time-to-Peak Untwist Rate (ms)**	**Deceleration Time (ms)**	
**RSD/minimum sample size**	57/36	14/3	18/4	

As observed from the Bland Altman plots, excellent intra-subject repeatability was obtained for peak strain, strain-rate and deceleration time with low bias in the mean difference, narrow limits of repeatability and smaller limit of repeatability to mean value ratios. Moderate intra-subject repeatability is observed for E/CSR and E/LSR ratios, peak torsion and time to peak untwist rate with broader limits of repeatability and moderate limit of repeatability to mean value ratios; however maintaining low bias in the mean difference. Poor intra-subject repeatability was observed for peak untwist rate and strain dyssynchrony with the limits of repeatability being of the order of the mean of the measurements.

As seen in Tables 2 and 3, the concordance correlation coefficient was excellent for peak strain and strain-rates (0.7–0.97), moderate for peak torsion, peak untwist rate, E/CSR and E/LSR ratios, and deceleration time (0.45–0.83) and were poor for strain dyssynchrony and time to peak untwist rate (0.1–0.34). Combining the results, peak strain and strain-rate, E/CSR and E/LSR ratios and deceleration time demonstrated moderate-to-good repeatability in measurements.

Results obtained from inter-subject variability studies are shown in Table 4, indicating low variability in peak strain and systolic strain-rate (relative standard deviation (RSD) < 25%) and minimum sample size (< 25) needed to achieve detection of a 15% change in effect size with power of 80% at a significance level of P < 0.05. Although the sample size needed for peak torsion and strain dyssynchrony was also < 25, the relative standard deviations in these parameters were higher (~30–50%).

Results of inter-subject variability studies listed in Table 5 reveal least variability in diastolic timing parameters (relative standard deviation (RSD) < 20%) and minimum sample size (<5). Diastolic E/CSR and E/LSR ratios had higher relative standard deviation (25–43%), but the minimum sample size needed was less than 21. Diastolic strain-rates and peak untwist rate were increasingly variable within the imaged cohort and required a larger sample size to achieve detection of a 15% change in effect size.

In our study, the biomarkers that had both good repeatability and low sample size were peak systolic strain, average systolic strain-rate, diastolic E/CSR and E/LSR ratios, and deceleration time.

## Discussion

In this work, a comprehensive characterization of LV function in naïve NHPs using MR imaging was conducted. The imaging parameters in commercially available MR imaging pulse sequences were optimized to perform regional quantification of cardiac function and flow in these animals. While several aspects of the imaging pulse sequences used can be further modified to improve image quality and strain/flow quantification, our approach of using standardized pulse sequences lends itself towards favorable repeatability of image quality across multiple study sites, provided the magnet strength and gradient hardware is comparable. In addition, statistical analyses of both intra-subject repeatability and inter-subject variability of cardiac MRI measurements that could be used as quantitative cardiac functional biomarkers are also presented. The inter-subject variability may or may not depend on the intra-subject repeatability of that biomarker. For example, we find that the relative standard deviations within the cohort was low for time to peak untwist rate, while the concordance correlation coefficient for this biomarker was poor. On the contrary, we find that diastolic strain-rates demonstrated high variability within the imaged cohort, but the test-retest repeatability was moderately good. Thus trade-offs between these two types of statistical analyses need to be considered in the ultimate choice of biomarkers. The ideal biomarker would demonstrate relatively high repeatability for test-retest measurements and low variability within the imaged cohort.

The non-invasive biomarkers evaluated in our study can be used to comprehensively characterize both systolic and diastolic function in normal as well as diseased hearts. Peak strain and systolic strain-rate (both identified as reproducible and reliable imaging markers in this study) are known to be altered significantly during systolic dysfunction [53]. Similarly, E/CSR and E/LSR ratios and deceleration time (again identified as robust diastolic biomarkers in this study) have been demonstrated to play an important role in identifying early diastolic dysfunction [54,55]. It has been shown that diastolic dysfunction in hypertrophied hearts is typically associated with elevated E/LSR ratios, and altered filling dynamics with eventual shortened deceleration times [54,55]. Quantitatively, it was reported, for example, that patients with diabetes mellitus exhibit around 15% lower peak circumferential strain, 22% lower peak longitudinal strain, 10–15% lower systolic strain-rates, 30–35% lower diastolic strain-rates than healthy control subjects [56,57]. An 18% decrease in mitral inflow velocity was also observed in these patients as a consequence of diastolic dysfunction [58]. Other studies conducted in patients newly diagnosed with hypertension demonstrate that a 15–30% decrease in peak circumferential strain is observed in these subjects [59,60]. Patients with non-obstructive hypertrophic cardiomyopathy also exhibit a 17% decrease in peak circumferential strain, 50% decrease in peak longitudinal strain, 10–20% change in systolic strain-rates and 25–30% change in diastolic strain-rates [10,61]. Our sample size computations were based on a 15% effect, which is lower than most reported changes observed in diseased patients. As a result, we are confident that the non-invasive biomarkers presented here have great potential in detecting early subtle changes in cardiac function (due to surgical interventional cardiac models of disease or pharmaceutical interventions) and tracking these changes longitudinally in pre-clinical NHP cardiac studies.

We observe similar functional characteristics in NHPs as has been previously observed in humans [52]. For example, our data support a predominant LV dimension change during early systole, followed by a more active torsion-based pumping after peak aortic outflow velocity has been achieved. During diastole, about 65% of the untwisting occurs during early diastole until the rapid filling is completed, while the remaining 35% occurs during the rest of diastole, likely to enable efficient filling during atrial kick. Previously, Jung et al. have found that early systolic rotation behavior in mice is opposite in direction to humans [62], while we observe clockwise rotation in all slices during early systole in NHPs, similar to humans (see Fig 3F). Studies conducted in naive dogs also reveal rotational characteristics similar to humans [63]. Additionally, we found no regional changes between axial segments in longitudinal strain in NHPs. Although the basal longitudinal strain is higher as compared to other segments as in humans, it is not statistically significant. In humans, there is a predominantly higher longitudinal strain in the base as well as the lateral wall, while in mice it is observed that the longitudinal strain is the highest in the opposing septal wall [62]. Peak circumferential strain in the base was found to be significantly lower in NHPs, a similar characteristic to what has been previously observed in humans and dogs [64,65]. To summarize, the NHP cardiac mechanics more closely resembled the human heart and naive dogs in comparison to mice. Thus, the NHP model may play a significant role in developing functional phenotypes translatable to human cardiac diseases.

Finally, we noticed that there are limitations in the current study. The HARP filter was designed to maximally capture the frequency components of interest, while minimizing interference from neighboring harmonic and dc peaks. The maximum strain resolution that we are able to achieve using this filter is 3 mm × 3 mm in-plane. To detect small changes in strain that are also localized in small regions, a higher strain resolution may be desirable. This can be achieved using the CSPAMM/CANSEL imaging technique [66,67], combined with larger HARP filter diameters. This approach will, however, require specialized imaging pulse sequences, not widely available on most scanners to date.

## Conclusions

An MR imaging protocol and data analysis platform has been developed to successfully provide a comprehensive characterization of NHP cardiac function. The imaging framework enables robust non-invasive quantification of primary systolic and diastolic functional imaging biomarkers. The intra-subject and inter-subject statistical analyses reported in our work strongly support the application of selected functional imaging biomarkers in future pre-clinical pharmacological studies using NHP models of cardiac disease.

## Supporting Information

S1 File Tables listing the computed values of all regional biomarkers for each of the imaged animals. Computed values for peak circumferential strain in three short-axis slices (apical, mid and basal) and sub-regions as defined in Fig 3. (**Table A**). Computed values for systolic circumferential strain rate in three short-axis slices (apical, mid and basal) and sub-regions as defined in Fig 3. (**Table B**). Computed values for peak longitudinal strain in two long-axis slices (2 chamber and 4 chamber) and sub-regions as defined in Fig 3. (**Table C**). Computed values for systolic longitudinal strain rate in two long-axis slices (2 chamber and 4 chamber) and sub-regions as defined in Fig 3. (**Table D**). Computed values for peak base-apex, mid-apex, and base-mid torsion (**Table E**). Computed values for circumferential strain dyssynchrony in three short-axis slices (apical, mid and basal) (**Table F**). Computed values for longitudinal strain dyssynchrony for three levels (apical, mid and basal) (**Table G**). Computed values for diastolic circumferential strain rate in three short-axis slices (apical, mid and basal) and sub-regions as defined in Fig 3. (**Table H**). Computed values for diastolic longitudinal strain rate in two long-axis slices (2 chamber and 4 chamber) and sub-regions as defined in Fig 3. (**Table I**). Computed values for the diastolic E/CSR ratio in three short-axis slices (apical, mid and basal) and sub-regions as defined in Fig 3. (**Table J**). Computed values for the diastolic E/LSR ratio in two long-axis slices (2 chamber and 4 chamber) and sub-regions as defined in Fig 3. (**Table K**). Computed values for peak base-apex, mid-apex, and base-mid untwist rate (**Table L**). Computed values for time to peak base-apex, mid-apex, and base-mid untwist rate (**Table M**). Computed values for global deceleration time (**Table N**).
(DOCX)
