# Supplementary material for: Characterization of Regional Left Ventricular Function in Nonhuman Primates Using Magnetic Resonance Imaging Biomarkers: A Test-Retest Repeatability and Inter-Subject Variability Study
Source: PLoS One. 2015 May 26;10(5):e0127947. doi: 10.1371/journal.pone.0127947 (PMC4444127; doi:10.1371/journal.pone.0127947)
Supplement: S1 File — Computed values for peak circumferential strain in three short-axis slices (apical, mid and basal) and sub-regions as defined in Fig 3. (Table A). Computed values for systolic circumferential strain rate in three short-axis slices (apical, mid and basal) and sub-regions as defined in Fig 3. (Table B). Computed values for peak longitudinal strain in two long-axis slices (2 chamber and 4 chamber) and sub-regions as defined in Fig 3. (Table C). Computed values for systolic longitudinal strain rate in two long-axis slices (2 chamber and 4 chamber) and sub-regions as defined in Fig 3. (Table D). Computed values for peak base-apex, mid-apex, and base-mid torsion (Table E). Computed values for circumferential strain dyssynchrony in three short-axis slices (apical, mid and basal) (Table F). Computed values for longitudinal strain dyssynchrony for three levels (apical, mid and basal) (Table G). Computed values for diastolic circumferential strain rate in three short-axis slices (apical, mid and basal) and sub-regions as defined in Fig 3. (Table H). Computed values for diastolic longitudinal strain rate in two long-axis slices (2 chamber and 4 chamber) and sub-regions as defined in Fig 3. (Table I). Computed values for the diastolic E/CSR ratio in three short-axis slices (apical, mid and basal) and sub-regions as defined in Fig 3. (Table J). Computed values for the diastolic E/LSR ratio in two long-axis slices (2 chamber and 4 chamber) and sub-regions as defined in Fig 3. (Table K). Computed values for peak base-apex, mid-apex, and base-mid untwist rate (Table L). Computed values for time to peak base-apex, mid-apex, and base-mid untwist rate (Table M). Computed values for global deceleration time (Table N). (DOCX) [file pone.0127947.s001.docx]

**S1 File. Tables listing the computed values of all regional biomarkers for each of the imaged animals.**

**Table A. Peak circumferential strain**

|  |  | **Animal 1** | | **Animal 2** | | **Animal 3** | | **Animal 4** | | **Animal 5** | | **Animal 6** | | **Animal 7** | | **Animal 8** | |
| --- | --- | --- | --- | --- | --- | --- | --- | --- | --- | --- | --- | --- | --- | --- | --- | --- | --- |
|  |  | **Test** | **Retest** | **Test** | **Retest** | **Test** | **Retest** | **Test** | **Retest** | **Test** | **Retest** | **Test** | **Retest** | **Test** | **Retest** | **Test** | **Retest** |
| **Apical** | 1 | -17.75 | -18.57 | -24.55 | -24.91 | -16.02 | -18.13 | -17.44 | -18.27 | -18.71 | -17.27 | -22.15 | -21.16 | -18.98 | -19.31 | -18.30 | -19.27 |
|  | 2 | -17.80 | -18.34 | -24.86 | -28.01 | -17.22 | -21.15 | -18.59 | -15.59 | -17.43 | -16.93 | -22.04 | -21.31 | -18.95 | -22.17 | -18.10 | -21.70 |
|  | 3 | -16.28 | -18.23 | -15.83 | -19.09 | -24.70 | -24.44 | -15.91 | -17.43 | -13.79 | -15.01 | -19.19 | -21.63 | -18.18 | -20.22 | -16.69 | -18.11 |
|  | 4 | -16.22 | -18.30 | -15.96 | -18.61 | -18.36 | -19.19 | -19.18 | -18.00 | -15.85 | -14.91 | -19.22 | -17.44 | -20.04 | -20.85 | -22.70 | -21.81 |
| **Mid** | 1 | -18.44 | -15.22 | -23.04 | -20.78 | -20.23 | -19.61 | -17.59 | -18.20 | -13.60 | -13.67 | -22.39 | -21.03 | -17.05 | -20.22 | -19.54 | -22.37 |
|  | 2 | -17.88 | -16.78 | -21.02 | -24.14 | -18.43 | -19.51 | -16.83 | -14.87 | -18.69 | -16.96 | -22.58 | -20.28 | -15.80 | -16.60 | -20.28 | -23.15 |
|  | 3 | -17.32 | -13.04 | -23.11 | -21.79 | -19.02 | -18.49 | -15.60 | -15.52 | -17.34 | -14.10 | -23.27 | -19.47 | -21.12 | -21.61 | -23.34 | -23.14 |
|  | 4 | -18.14 | -19.17 | -21.39 | -26.34 | -18.76 | -18.67 | -16.82 | -16.83 | -15.00 | -13.81 | -22.33 | -23.40 | -17.78 | -18.30 | -16.13 | -19.72 |
|  | 5 | -18.36 | -18.56 | -27.97 | -23.71 | -23.18 | -21.40 | -18.79 | -17.84 | -14.76 | -14.98 | -20.57 | -22.63 | -19.89 | -24.37 | -20.97 | -21.02 |
|  | 6 | -19.84 | -17.58 | -19.09 | -20.21 | -16.37 | -16.94 | -17.02 | -15.69 | -15.28 | -13.47 | -19.22 | -20.02 | -17.00 | -17.71 | -15.05 | -15.09 |
| **Basal** | 1 | -13.33 | -14.68 | -22.81 | -20.02 | -16.99 | -18.16 | -12.33 | -9.87 | -15.58 | -12.23 | -18.20 | -15.49 | -12.97 | -15.06 | -18.16 | -16.90 |
|  | 2 | -13.32 | -14.64 | -19.47 | -18.12 | -16.47 | -18.94 | -12.05 | -13.53 | -9.82 | -12.49 | -16.06 | -14.01 | -13.41 | -14.45 | -18.11 | -17.94 |
|  | 3 | -14.81 | -13.94 | -22.13 | -20.71 | -15.40 | -16.72 | -14.43 | -12.30 | -15.39 | -14.89 | -14.95 | -12.57 | -13.11 | -16.03 | -14.10 | -14.48 |
|  | 4 | -14.33 | -14.56 | -17.00 | -19.24 | -17.44 | -19.04 | -12.25 | -12.00 | -13.16 | -11.40 | -19.76 | -17.92 | -12.76 | -14.73 | -16.62 | -16.19 |
|  | 5 | -18.06 | -18.25 | -20.85 | -21.02 | -20.45 | -17.73 | -15.16 | -14.45 | -14.70 | -15.90 | -15.95 | -13.09 | -13.20 | -15.08 | -17.27 | -12.49 |
|  | 6 | -10.45 | -11.44 | -16.46 | -17.84 | -15.29 | -15.63 | -8.81 | -10.89 | -9.49 | -8.09 | -14.16 | -9.85 | -12.42 | -13.81 | -13.17 | -17.85 |

**Table B. Systolic circumferential strain rate**

|  |  | **Animal 1** | | **Animal 2** | | **Animal 3** | | **Animal 4** | | **Animal 5** | | **Animal 6** | | **Animal 7** | | **Animal 8** | |
| --- | --- | --- | --- | --- | --- | --- | --- | --- | --- | --- | --- | --- | --- | --- | --- | --- | --- |
|  |  | **Test** | **Retest** | **Test** | **Retest** | **Test** | **Retest** | **Test** | **Retest** | **Test** | **Retest** | **Test** | **Retest** | **Test** | **Retest** | **Test** | **Retest** |
| **Apical** | 1 | -80.56 | -72.79 | -102.16 | -99.90 | -119.74 | -121.07 | -119.52 | -122.08 | -114.89 | -99.63 | -94.73 | -95.58 | -80.83 | -81.15 | -80.15 | -83.48 |
|  | 2 | -82.41 | -79.74 | -103.77 | -112.38 | -125.52 | -129.70 | -126.15 | -119.03 | -111.63 | -101.35 | -98.85 | -96.61 | -79.43 | -89.95 | -81.29 | -92.34 |
|  | 3 | -69.41 | -67.63 | -67.58 | -83.74 | -143.17 | -139.28 | -115.90 | -121.31 | -89.52 | -91.82 | -87.30 | -95.24 | -80.15 | -84.77 | -75.85 | -79.19 |
|  | 4 | -77.32 | -76.89 | -65.83 | -77.37 | -128.22 | -128.95 | -128.38 | -124.63 | -96.18 | -93.11 | -90.26 | -84.71 | -83.84 | -89.55 | -96.42 | -91.72 |
| **Mid** | 1 | -74.26 | -58.82 | -97.34 | -87.73 | -130.38 | -125.43 | -121.19 | -124.00 | -87.08 | -86.79 | -97.64 | -94.18 | -78.27 | -85.81 | -84.72 | -92.52 |
|  | 2 | -61.99 | -76.67 | -90.20 | -105.03 | -127.04 | -129.60 | -119.12 | -115.99 | -104.70 | -99.79 | -99.23 | -92.56 | -70.95 | -74.25 | -89.43 | -96.26 |
|  | 3 | -76.59 | -58.25 | -96.94 | -91.05 | -132.01 | -127.25 | -115.75 | -120.66 | -99.35 | -91.50 | -101.52 | -88.39 | -88.94 | -89.94 | -99.87 | -96.54 |
|  | 4 | -79.36 | -80.90 | -92.44 | -111.25 | -127.31 | -123.65 | -119.45 | -121.47 | -93.87 | -90.35 | -97.89 | -101.48 | -76.26 | -82.12 | -70.71 | -85.99 |
|  | 5 | -77.45 | -78.59 | -116.99 | -101.74 | -138.45 | -132.11 | -123.52 | -122.80 | -92.69 | -93.13 | -91.76 | -98.06 | -83.21 | -100.69 | -89.72 | -88.24 |
|  | 6 | -85.74 | -74.36 | -79.85 | -82.50 | -121.74 | -120.64 | -122.59 | -119.67 | -100.19 | -92.50 | -88.15 | -91.23 | -75.44 | -80.14 | -72.77 | -70.15 |
| **Basal** | 1 | -58.22 | -56.90 | -93.79 | -122.82 | -121.98 | -121.16 | -98.37 | -87.81 | -86.05 | -55.67 | -75.61 | -68.74 | -71.57 | -72.02 | -80.56 | -72.79 |
|  | 2 | -63.18 | -74.48 | -92.87 | -126.19 | -121.81 | -131.13 | -106.75 | -108.01 | -73.80 | -58.82 | -74.89 | -71.75 | -71.43 | -68.57 | -82.41 | -79.74 |
|  | 3 | -69.99 | -59.42 | -95.32 | -130.57 | -118.01 | -124.08 | -108.07 | -102.10 | -91.41 | -66.35 | -77.55 | -63.44 | -65.85 | -71.77 | -69.41 | -67.63 |
|  | 4 | -69.47 | -77.53 | -100.00 | -126.38 | -121.30 | -127.25 | -106.58 | -100.00 | -87.26 | -57.64 | -89.35 | -84.17 | -70.90 | -70.23 | -77.32 | -76.89 |
|  | 5 | -85.01 | -82.12 | -128.78 | -131.61 | -130.60 | -122.12 | -109.83 | -108.05 | -64.82 | -69.93 | -80.41 | -67.99 | -68.55 | -73.42 | -74.26 | -58.82 |
|  | 6 | -43.56 | -44.95 | -113.24 | -121.59 | -114.98 | -112.43 | -83.39 | -96.09 | -48.81 | -46.30 | -80.51 | -57.36 | -58.89 | -64.52 | -61.99 | -76.67 |

**Table C. Peak longitudinal strain**

|  |  | **Animal 1** | | **Animal 2** | | **Animal 3** | | **Animal 4** | | **Animal 5** | | **Animal 6** | | **Animal 7** | | **Animal 8** | |
| --- | --- | --- | --- | --- | --- | --- | --- | --- | --- | --- | --- | --- | --- | --- | --- | --- | --- |
|  |  | **Test** | **Retest** | **Test** | **Retest** | **Test** | **Retest** | **Test** | **Retest** | **Test** | **Retest** | **Test** | **Retest** | **Test** | **Retest** | **Test** | **Retest** |
| **Apical** | **2ch3** | -13.94 | -14.59 | -15.51 | -14.56 | -7.42 | -5.69 | -13.53 | -14.24 | -13.74 | -14.45 | -17.23 | -13.71 | -11.00 | -11.11 | -8.65 | -13.49 |
|  | **2ch6** | -14.95 | -13.03 | -20.13 | -24.95 | -14.84 | -18.55 | -11.22 | -11.61 | -18.42 | -13.38 | -9.82 | -10.28 | -18.25 | -19.55 | -19.05 | -16.29 |
|  | **4ch3** | -14.00 | -13.90 | -22.44 | -23.49 | -12.92 | -14.14 | -9.28 | -12.07 | -14.50 | -15.37 | -17.30 | -13.10 | -17.24 | -13.85 | -9.48 | -14.06 |
|  | **4ch6** | -11.85 | -18.15 | -21.34 | -22.24 | -13.51 | -14.46 | -10.57 | -12.48 | -16.48 | -14.12 | -14.09 | -14.89 | -17.05 | -15.13 | -16.40 | -11.42 |
| **Mid** | **2ch2** | -12.37 | -13.16 | -19.30 | -19.80 | -15.41 | -14.28 | -15.68 | -14.89 | -15.93 | -16.99 | -19.77 | -15.44 | -16.62 | -17.29 | -13.12 | -15.89 |
|  | **2ch5** | -11.89 | -14.35 | -19.60 | -22.76 | -16.05 | -13.79 | -19.38 | -18.21 | -11.16 | -11.19 | -16.14 | -13.74 | -9.90 | -15.48 | -15.33 | -15.58 |
|  | **4ch2** | -14.20 | -12.77 | -18.33 | -18.02 | -12.66 | -10.94 | -16.05 | -13.60 | -11.35 | -8.16 | -17.26 | -12.99 | -12.64 | -16.84 | -15.46 | -13.18 |
|  | **4ch5** | -11.81 | -14.45 | -17.02 | -19.78 | -11.39 | -15.08 | -12.66 | -13.94 | -9.20 | -11.62 | -13.88 | -14.24 | -14.48 | -15.86 | -18.33 | -14.28 |
| **Basal** | **2ch1** | -8.87 | -11.06 | -16.73 | -15.08 | -7.01 | -8.26 | -13.56 | -13.13 | -13.53 | -11.89 | -13.90 | -15.25 | -13.50 | -16.09 | -10.16 | -9.83 |
|  | **2ch4** | -13.89 | -11.28 | -18.79 | -22.17 | -18.62 | -19.39 | -20.38 | -19.60 | -16.01 | -12.35 | -17.36 | -19.93 | -14.82 | -16.28 | -19.08 | -19.93 |
|  | **4ch1** | -14.09 | -11.45 | -14.85 | -16.30 | -17.79 | -15.62 | -11.14 | -13.79 | -12.59 | -15.07 | -14.58 | -14.86 | -10.58 | -9.14 | -13.25 | -14.90 |
|  | **4ch4** | -16.84 | -14.58 | -24.78 | -25.41 | -17.76 | -21.57 | -17.52 | -19.14 | -14.36 | -13.40 | -21.13 | -16.92 | -20.72 | -21.39 | -20.51 | -18.84 |

**Table D. Systolic longitudinal strain rate**

|  |  | **Animal 1** | | **Animal 2** | | **Animal 3** | | **Animal 4** | | **Animal 5** | | **Animal 6** | | **Animal 7** | | **Animal 8** | |
| --- | --- | --- | --- | --- | --- | --- | --- | --- | --- | --- | --- | --- | --- | --- | --- | --- | --- |
|  |  | **Test** | **Retest** | **Test** | **Retest** | **Test** | **Retest** | **Test** | **Retest** | **Test** | **Retest** | **Test** | **Retest** | **Test** | **Retest** | **Test** | **Retest** |
| **Apical** | **2ch3** | -58.24 | -52.05 | -54.99 | -49.62 | -38.95 | -28.84 | -54.90 | -61.10 | -55.78 | -59.29 | -77.09 | -58.33 | -44.04 | -42.12 | -42.25 | -56.96 |
|  | **2ch6** | -59.57 | -41.40 | -73.21 | -87.67 | -61.04 | -67.94 | -42.87 | -52.07 | -65.65 | -57.77 | -61.55 | -43.66 | -71.00 | -74.13 | -76.01 | -65.47 |
|  | **4ch3** | -41.28 | -46.30 | -82.80 | -86.63 | -54.83 | -55.05 | -37.26 | -52.00 | -52.50 | -55.95 | -73.09 | -58.37 | -69.37 | -53.78 | -42.75 | -55.49 |
|  | **4ch6** | -46.81 | -64.59 | -79.50 | -80.13 | -55.53 | -56.34 | -37.20 | -50.42 | -57.66 | -51.81 | -53.79 | -57.48 | -62.79 | -54.52 | -62.98 | -52.38 |
| **Mid** | **2ch2** | -57.56 | -48.17 | -76.82 | -77.50 | -68.21 | -59.07 | -64.70 | -62.28 | -58.02 | -61.71 | -88.10 | -72.19 | -69.70 | -67.79 | -58.72 | -64.68 |
|  | **2ch5** | -45.91 | -57.86 | -77.78 | -80.83 | -61.45 | -50.86 | -69.70 | -75.14 | -50.02 | -46.81 | -73.11 | -57.58 | -40.63 | -62.80 | -65.06 | -65.27 |
|  | **4ch2** | -56.04 | -40.87 | -69.45 | -67.13 | -55.47 | -46.08 | -65.76 | -59.73 | -44.42 | -30.19 | -70.07 | -57.18 | -56.93 | -67.85 | -66.33 | -53.48 |
|  | **4ch5** | -39.20 | -49.81 | -57.86 | -63.99 | -47.16 | -51.43 | -47.34 | -55.05 | -32.44 | -40.50 | -51.72 | -61.37 | -59.11 | -58.90 | -73.17 | -57.72 |
| **Basal** | **2ch1** | -43.56 | -36.76 | -69.45 | -61.59 | -38.19 | -48.22 | -58.34 | -58.69 | -47.73 | -54.10 | -69.07 | -65.50 | -63.51 | -67.36 | -51.17 | -46.86 |
|  | **2ch4** | -49.06 | -45.10 | -66.39 | -74.90 | -65.42 | -68.56 | -73.74 | -77.02 | -53.72 | -45.02 | -64.07 | -78.77 | -58.46 | -68.98 | -75.27 | -74.71 |
|  | **4ch1** | -52.19 | -42.92 | -57.92 | -64.30 | -66.11 | -62.62 | -53.60 | -56.88 | -50.76 | -58.47 | -62.24 | -58.09 | -54.74 | -42.96 | -51.68 | -55.56 |
|  | **4ch4** | -60.47 | -51.13 | -90.78 | -95.03 | -69.71 | -80.91 | -66.37 | -76.37 | -53.77 | -47.38 | -81.47 | -69.96 | -88.65 | -81.29 | -82.72 | -76.37 |

**Table E. Peak torsion**

|  | **Animal 1** | | **Animal 2** | | **Animal 3** | | **Animal 4** | | **Animal 5** | | **Animal 6** | | **Animal 7** | | **Animal 8** | |
| --- | --- | --- | --- | --- | --- | --- | --- | --- | --- | --- | --- | --- | --- | --- | --- | --- |
|  | **Test** | **Retest** | **Test** | **Retest** | **Test** | **Retest** | **Test** | **Retest** | **Test** | **Retest** | **Test** | **Retest** | **Test** | **Retest** | **Test** | **Retest** |
| **Base-Apex** | -4.72 | -6.41 | -7.56 | -8.14 | -5.36 | -6.13 | -2.88 | -2.75 | -3.23 | -3.19 | -7.25 | -3.61 | -3.55 | -4.57 | -3.79 | -3.33 |
| **Mid-Apex** | -1.86 | -3.35 | -4 | -4.4 | -3.25 | -3.66 | -1.9 | -1.33 | -2 | -1.75 | -4.57 | -2.17 | -2.06 | -2.4 | -2.14 | -1.76 |
| **Base-Mid** | -3 | -3.48 | -4.06 | -4.31 | -2.33 | -3 | -1.69 | -1.57 | -1.31 | -1.64 | -2.92 | -1.85 | -1.84 | -2.38 | -1.7 | -1.71 |

**Table F. Circumferential strain dyssynchrony**

|  | **Animal 1** | | **Animal 2** | | **Animal 3** | | **Animal 4** | | **Animal 5** | | **Animal 6** | | **Animal 7** | | **Animal 8** | |
| --- | --- | --- | --- | --- | --- | --- | --- | --- | --- | --- | --- | --- | --- | --- | --- | --- |
|  | **Test** | **Retest** | **Test** | **Retest** | **Test** | **Retest** | **Test** | **Retest** | **Test** | **Retest** | **Test** | **Retest** | **Test** | **Retest** | **Test** | **Retest** |
| **Apex** | 7.07 | 4.79 | 7.07 | 8.54 | 10.80 | 6.45 | 4.79 | 11.09 | 11.09 | 10.80 | 6.29 | 6.45 | 6.29 | 6.45 | 4.79 | 2.89 |
| **Mid** | 3.76 | 8.61 | 5.48 | 7.53 | 6.32 | 5.24 | 3.16 | 6.65 | 12.14 | 9.31 | 5.85 | 3.16 | 5.48 | 6.83 | 6.06 | 6.06 |
| **Base** | 20.43 | 35.45 | 20.74 | 15.38 | 14.14 | 22.89 | 34.45 | 27.87 | 41.16 | 20.74 | 38.04 | 24.43 | 30.77 | 21.78 | 20.17 | 28.53 |

**Table G. Longitudinal strain dyssynchrony**

|  | **Animal 1** | | **Animal 2** | | **Animal 3** | | **Animal 4** | | **Animal 5** | | **Animal 6** | | **Animal 7** | | **Animal 8** | |
| --- | --- | --- | --- | --- | --- | --- | --- | --- | --- | --- | --- | --- | --- | --- | --- | --- |
|  | **Test** | **Retest** | **Test** | **Retest** | **Test** | **Retest** | **Test** | **Retest** | **Test** | **Retest** | **Test** | **Retest** | **Test** | **Retest** | **Test** | **Retest** |
| **Apex** | 27.20 | 19.31 | 18.87 | 14.43 | 31.62 | 34.97 | 27.99 | 17.32 | 12.25 | 12.50 | 26.89 | 14.93 | 19.74 | 14.14 | 12.25 | 25.82 |
| **Mid** | 11.90 | 24.15 | 13.77 | 13.54 | 10.31 | 16.52 | 22.87 | 16.52 | 20.56 | 9.46 | 20.97 | 7.50 | 12.25 | 10.80 | 2.50 | 4.79 |
| **Base** | 18.48 | 24.28 | 9.13 | 11.90 | 17.97 | 18.48 | 17.08 | 14.72 | 11.09 | 20.97 | 31.72 | 8.54 | 13.54 | 2.50 | 17.80 | 13.15 |

**Table H. Diastolic circumferential strain rate**

|  |  | **Animal 1** | | **Animal 2** | | **Animal 3** | | **Animal 4** | | **Animal 5** | | **Animal 6** | | **Animal 7** | | **Animal 8** | |
| --- | --- | --- | --- | --- | --- | --- | --- | --- | --- | --- | --- | --- | --- | --- | --- | --- | --- |
|  |  | **Test** | **Retest** | **Test** | **Retest** | **Test** | **Retest** | **Test** | **Retest** | **Test** | **Retest** | **Test** | **Retest** | **Test** | **Retest** | **Test** | **Retest** |
| **Apical** | 1 | 139.31 | 139.74 | 161.43 | 131.52 | 104.46 | 124.65 | 90.33 | 82.40 | 87.95 | 91.49 | 118.27 | 94.91 | 96.86 | 92.30 | 105.96 | 96.86 |
|  | 2 | 150.18 | 146.08 | 153.23 | 187.74 | 111.74 | 153.53 | 97.16 | 77.32 | 93.52 | 92.91 | 128.70 | 100.65 | 91.87 | 87.61 | 133.56 | 143.79 |
|  | 3 | 138.21 | 150.89 | 105.52 | 120.02 | 146.24 | 130.85 | 71.12 | 93.97 | 101.02 | 97.51 | 101.17 | 107.40 | 84.83 | 87.00 | 94.90 | 118.10 |
|  | 4 | 148.37 | 163.31 | 112.66 | 117.04 | 138.61 | 132.48 | 128.88 | 107.62 | 93.39 | 93.47 | 113.15 | 96.92 | 98.45 | 106.07 | 131.87 | 142.74 |
| **Mid** | 1 | 109.33 | 157.21 | 158.41 | 132.09 | 122.50 | 124.83 | 96.58 | 119.28 | 87.45 | 90.53 | 104.16 | 117.45 | 92.99 | 105.58 | 102.94 | 126.19 |
|  | 2 | 164.49 | 129.71 | 129.30 | 137.62 | 113.27 | 118.57 | 76.38 | 79.65 | 107.24 | 104.79 | 131.67 | 101.53 | 66.34 | 73.30 | 120.47 | 136.17 |
|  | 3 | 135.33 | 90.01 | 125.29 | 142.31 | 120.12 | 102.89 | 87.85 | 97.82 | 109.31 | 84.95 | 139.57 | 109.53 | 104.26 | 96.54 | 132.96 | 118.82 |
|  | 4 | 160.93 | 159.05 | 130.92 | 156.16 | 119.78 | 117.56 | 93.09 | 103.26 | 82.13 | 81.87 | 185.29 | 123.16 | 89.65 | 91.18 | 91.43 | 134.06 |
|  | 5 | 152.43 | 107.03 | 149.90 | 149.11 | 126.18 | 124.91 | 96.94 | 90.75 | 84.39 | 95.58 | 114.50 | 117.94 | 84.40 | 115.48 | 124.23 | 113.15 |
|  | 6 | 180.60 | 145.53 | 140.82 | 155.71 | 109.26 | 106.31 | 122.67 | 86.61 | 67.74 | 82.84 | 105.97 | 103.37 | 93.03 | 94.91 | 104.67 | 108.89 |
| **Basal** | 1 | 73.15 | 102.05 | 109.96 | 184.87 | 94.96 | 112.53 | 66.78 | 43.76 | 103.44 | 62.38 | 78.98 | 71.74 | 55.19 | 81.05 | 101.42 | 117.03 |
|  | 2 | 57.86 | 51.15 | 99.04 | 102.98 | 96.52 | 117.66 | 87.64 | 84.58 | 29.74 | 64.32 | 92.46 | 59.16 | 46.69 | 86.23 | 115.77 | 114.70 |
|  | 3 | 99.33 | 92.57 | 145.51 | 159.03 | 102.45 | 95.89 | 52.35 | 70.60 | 66.90 | 74.94 | 83.95 | 62.87 | 53.92 | 83.78 | 102.45 | 118.09 |
|  | 4 | 47.65 | 40.56 | 130.00 | 131.51 | 104.23 | 103.95 | 36.10 | 30.00 | 41.32 | 57.37 | 96.65 | 87.82 | 36.81 | 81.81 | 107.69 | 77.81 |
|  | 5 | 82.20 | 94.72 | 139.94 | 160.05 | 104.71 | 113.62 | 77.03 | 50.08 | 50.67 | 82.11 | 96.52 | 44.85 | 67.21 | 75.17 | 122.47 | 111.93 |
|  | 6 | 74.86 | 75.14 | 146.23 | 171.86 | 111.34 | 111.24 | 59.33 | 65.08 | 41.43 | 59.38 | 48.35 | 55.54 | 86.55 | 97.55 | 108.54 | 102.17 |

**Table I. Diastolic longitudinal strain rate**

|  |  | **Animal 1** | | **Animal 2** | | **Animal 3** | | **Animal 4** | | **Animal 5** | | **Animal 6** | | **Animal 7** | | **Animal 8** | |
| --- | --- | --- | --- | --- | --- | --- | --- | --- | --- | --- | --- | --- | --- | --- | --- | --- | --- |
|  |  | **Test** | **Retest** | **Test** | **Retest** | **Test** | **Retest** | **Test** | **Retest** | **Test** | **Retest** | **Test** | **Retest** | **Test** | **Retest** | **Test** | **Retest** |
| **Apical** | **2ch3** | 70.41 | 98.62 | 139.10 | 150.08 | 68.04 | 72.05 | 73.84 | 82.71 | 78.21 | 82.81 | 131.19 | 62.40 | 70.20 | 82.84 | 90.40 | 95.07 |
|  | **2ch6** | 70.06 | 98.00 | 134.09 | 243.84 | 90.05 | 127.04 | 66.74 | 62.27 | 88.57 | 60.64 | 49.03 | 58.58 | 91.73 | 109.79 | 119.56 | 98.53 |
|  | **4ch3** | 101.83 | 94.76 | 183.63 | 167.72 | 95.37 | 110.54 | 73.08 | 74.92 | 83.94 | 96.49 | 64.97 | 36.95 | 95.56 | 97.39 | 76.25 | 69.53 |
|  | **4ch6** | 82.24 | 72.51 | 157.45 | 151.11 | 91.54 | 81.56 | 84.47 | 80.69 | 96.15 | 78.22 | 90.94 | 48.36 | 94.00 | 89.76 | 85.28 | 53.17 |
| **Mid** | **2ch2** | 126.72 | 96.15 | 152.51 | 174.56 | 105.25 | 132.66 | 126.44 | 116.76 | 111.24 | 121.62 | 144.94 | 135.97 | 106.21 | 99.62 | 88.35 | 112.92 |
|  | **2ch5** | 84.20 | 96.19 | 121.65 | 144.02 | 90.71 | 88.82 | 101.66 | 105.57 | 56.45 | 77.17 | 93.31 | 77.52 | 76.17 | 93.40 | 85.16 | 90.70 |
|  | **4ch2** | 86.32 | 85.12 | 126.37 | 127.60 | 75.90 | 68.88 | 91.58 | 96.03 | 68.40 | 82.57 | 50.28 | 49.37 | 76.74 | 86.92 | 74.57 | 79.17 |
|  | **4ch5** | 97.39 | 86.95 | 167.77 | 201.54 | 79.51 | 103.01 | 71.11 | 78.10 | 93.27 | 91.44 | 100.15 | 54.98 | 64.55 | 115.85 | 118.79 | 116.62 |
| **Basal** | **2ch1** | 71.05 | 90.51 | 147.41 | 160.86 | 61.76 | 51.29 | 121.26 | 105.75 | 93.77 | 68.74 | 91.75 | 72.54 | 77.45 | 101.10 | 55.50 | 62.89 |
|  | **2ch4** | 68.63 | 110.52 | 134.77 | 146.39 | 58.05 | 80.63 | 73.63 | 80.34 | 103.94 | 100.48 | 108.92 | 55.78 | 73.64 | 97.01 | 107.04 | 135.07 |
|  | **4ch1** | 81.15 | 39.57 | 109.48 | 121.74 | 85.34 | 63.51 | 55.72 | 90.56 | 51.98 | 58.37 | 50.98 | 72.01 | 58.73 | 49.73 | 65.36 | 88.46 |
|  | **4ch4** | 67.68 | 69.06 | 110.30 | 136.73 | 78.46 | 96.11 | 82.34 | 85.42 | 85.55 | 75.44 | 102.11 | 81.53 | 78.61 | 89.08 | 89.58 | 88.87 |

**Table J. E/CSR**

|  |  | **Animal 1** | | **Animal 2** | | **Animal 3** | | **Animal 4** | | **Animal 5** | | **Animal 6** | | **Animal 7** | | **Animal 8** | |
| --- | --- | --- | --- | --- | --- | --- | --- | --- | --- | --- | --- | --- | --- | --- | --- | --- | --- |
|  |  | **Test** | **Retest** | **Test** | **Retest** | **Test** | **Retest** | **Test** | **Retest** | **Test** | **Retest** | **Test** | **Retest** | **Test** | **Retest** | **Test** | **Retest** |
| **Apical** | 1 | 2.54 | 3.01 | 1.19 | 1.68 | 2.95 | 2.61 | 3.31 | 3.05 | 2.73 | 3.53 | 2.96 | 3.90 | 3.17 | 3.32 | 3.35 | 3.61 |
|  | 2 | 2.36 | 2.88 | 1.25 | 1.17 | 2.76 | 2.12 | 3.08 | 3.25 | 2.56 | 3.47 | 2.72 | 3.68 | 3.34 | 3.50 | 2.66 | 2.43 |
|  | 3 | 2.56 | 2.79 | 1.82 | 1.84 | 2.11 | 2.48 | 4.20 | 2.68 | 2.37 | 3.31 | 3.46 | 3.45 | 3.61 | 3.52 | 3.74 | 2.96 |
|  | 4 | 2.39 | 2.58 | 1.71 | 1.88 | 2.23 | 2.45 | 2.32 | 2.34 | 2.57 | 3.45 | 3.09 | 3.82 | 3.11 | 2.89 | 2.69 | 2.45 |
| **Mid** | 1 | 3.24 | 2.68 | 1.21 | 1.67 | 2.52 | 2.60 | 3.09 | 2.11 | 2.74 | 3.56 | 3.36 | 3.15 | 3.30 | 2.90 | 3.45 | 2.77 |
|  | 2 | 2.15 | 3.24 | 1.49 | 1.60 | 2.72 | 2.74 | 3.91 | 3.16 | 2.24 | 3.08 | 2.66 | 3.65 | 4.62 | 4.18 | 2.95 | 2.57 |
|  | 3 | 2.62 | 4.67 | 1.53 | 1.55 | 2.57 | 3.16 | 3.40 | 2.57 | 2.19 | 3.80 | 2.51 | 3.38 | 2.94 | 3.18 | 2.67 | 2.94 |
|  | 4 | 2.20 | 2.64 | 1.47 | 1.41 | 2.58 | 2.76 | 3.21 | 2.43 | 2.92 | 3.94 | 1.89 | 3.01 | 3.42 | 3.36 | 3.88 | 2.61 |
|  | 5 | 2.32 | 3.93 | 1.28 | 1.48 | 2.45 | 2.60 | 3.08 | 2.77 | 2.84 | 3.37 | 3.05 | 3.14 | 3.63 | 2.65 | 2.86 | 3.09 |
|  | 6 | 1.96 | 2.89 | 1.36 | 1.42 | 2.82 | 3.06 | 2.44 | 2.90 | 3.54 | 3.89 | 3.30 | 3.58 | 3.30 | 3.23 | 3.39 | 3.21 |
| **Basal** | 1 | 4.84 | 4.12 | 1.75 | 1.19 | 3.25 | 2.89 | 4.47 | 5.75 | 2.32 | 5.17 | 4.43 | 5.16 | 5.55 | 3.78 | 3.50 | 2.99 |
|  | 2 | 6.12 | 8.22 | 1.94 | 2.14 | 3.20 | 2.76 | 3.41 | 2.97 | 8.06 | 5.01 | 3.78 | 6.26 | 6.57 | 3.56 | 3.07 | 3.05 |
|  | 3 | 3.57 | 4.54 | 1.32 | 1.39 | 3.01 | 3.39 | 5.71 | 3.56 | 3.58 | 4.30 | 4.16 | 5.89 | 5.69 | 3.66 | 3.47 | 2.96 |
|  | 4 | 7.43 | 10.37 | 1.48 | 1.68 | 2.96 | 3.13 | 8.28 | 8.38 | 5.80 | 5.62 | 3.62 | 4.21 | 8.33 | 3.75 | 3.30 | 4.49 |
|  | 5 | 4.31 | 4.44 | 1.37 | 1.38 | 2.95 | 2.86 | 3.88 | 5.02 | 4.73 | 3.93 | 3.62 | 8.25 | 4.56 | 4.08 | 2.90 | 3.12 |
|  | 6 | 4.73 | 5.60 | 1.31 | 1.28 | 2.77 | 2.92 | 5.04 | 3.86 | 5.79 | 5.43 | 7.23 | 6.66 | 3.54 | 3.14 | 3.27 | 3.42 |

**Table K. E/LSR**

|  |  | **Animal 1** | | **Animal 2** | | **Animal 3** | | **Animal 4** | | **Animal 5** | | **Animal 6** | | **Animal 7** | | **Animal 8** | |
| --- | --- | --- | --- | --- | --- | --- | --- | --- | --- | --- | --- | --- | --- | --- | --- | --- | --- |
|  |  | **Test** | **Retest** | **Test** | **Retest** | **Test** | **Retest** | **Test** | **Retest** | **Test** | **Retest** | **Test** | **Retest** | **Test** | **Retest** | **Test** | **Retest** |
| **Apical** | **2ch3** | 5.03 | 4.26 | 1.38 | 1.47 | 4.53 | 4.51 | 4.05 | 3.04 | 3.07 | 3.89 | 2.67 | 5.93 | 4.37 | 3.70 | 3.93 | 3.68 |
|  | **2ch6** | 5.06 | 4.29 | 1.43 | 0.90 | 3.43 | 2.56 | 4.48 | 4.04 | 2.71 | 5.32 | 7.13 | 6.32 | 3.34 | 2.79 | 2.97 | 3.55 |
|  | **4ch3** | 3.48 | 4.44 | 1.05 | 1.31 | 3.23 | 2.94 | 4.09 | 3.36 | 2.86 | 3.34 | 5.38 | 10.02 | 3.21 | 3.15 | 4.66 | 5.03 |
|  | **4ch6** | 4.31 | 5.80 | 1.22 | 1.46 | 3.37 | 3.99 | 3.54 | 3.12 | 2.49 | 4.12 | 3.84 | 7.65 | 3.26 | 3.42 | 4.16 | 6.57 |
| **Mid** | **2ch2** | 2.80 | 4.37 | 1.26 | 1.26 | 2.93 | 2.45 | 2.36 | 2.15 | 2.16 | 2.65 | 2.41 | 2.72 | 2.89 | 3.08 | 4.02 | 3.10 |
|  | **2ch5** | 4.21 | 4.37 | 1.58 | 1.53 | 3.40 | 3.66 | 2.94 | 2.38 | 4.25 | 4.18 | 3.75 | 4.77 | 4.03 | 3.28 | 4.17 | 3.85 |
|  | **4ch2** | 4.10 | 4.94 | 1.52 | 1.73 | 4.06 | 4.72 | 3.26 | 2.62 | 3.50 | 3.91 | 6.95 | 7.50 | 3.99 | 3.53 | 4.76 | 4.42 |
|  | **4ch5** | 3.64 | 4.84 | 1.15 | 1.09 | 3.88 | 3.16 | 4.20 | 3.22 | 2.57 | 3.53 | 3.49 | 6.73 | 4.75 | 2.65 | 2.99 | 3.00 |
| **Basal** | **2ch1** | 4.99 | 4.65 | 1.30 | 1.37 | 5.00 | 6.34 | 2.46 | 2.38 | 2.56 | 4.69 | 3.81 | 5.10 | 3.96 | 3.03 | 6.40 | 5.56 |
|  | **2ch4** | 5.16 | 3.81 | 1.43 | 1.51 | 5.31 | 4.03 | 4.06 | 3.13 | 2.31 | 3.21 | 3.21 | 6.64 | 4.16 | 3.16 | 3.32 | 2.59 |
|  | **4ch1** | 4.36 | 10.63 | 1.75 | 1.81 | 3.62 | 5.12 | 5.36 | 2.78 | 4.61 | 5.52 | 6.86 | 5.14 | 5.22 | 6.16 | 5.43 | 3.95 |
|  | **4ch4** | 5.23 | 6.09 | 1.74 | 1.61 | 3.93 | 3.38 | 3.63 | 2.94 | 2.80 | 4.27 | 3.42 | 4.54 | 3.90 | 3.44 | 3.96 | 3.93 |

**Table L. Peak untwist rate**

|  | **Animal 1** | | **Animal 2** | | **Animal 3** | | **Animal 4** | | **Animal 5** | | **Animal 6** | | **Animal 7** | | | **Animal 8** | | |
| --- | --- | --- | --- | --- | --- | --- | --- | --- | --- | --- | --- | --- | --- | --- | --- | --- | --- | --- |
|  | **Test** | **Retest** | **Test** | **Retest** | **Test** | **Retest** | **Test** | **Retest** | **Test** | **Retest** | **Test** | **Retest** | **Test** | **Retest** | **Test** | | **Retest** |  |
| **Base-Apex** | 50.59 | 73.57 | 90.92 | 117.88 | 45.59 | 89.95 | 25.75 | 30.92 | 25.77 | 41.53 | 90.63 | 35.51 | 26.88 | 48.92 | 40.88 | | 32.99 |  |
| **Mid-Apex** | 31.14 | 55.23 | 65.22 | 73.96 | 31.75 | 56.85 | 34.05 | 11.82 | 20.89 | 25.72 | 78.6 | 29.55 | 22.23 | 37.61 | 35.31 | | 18.31 |  |
| **Base-Mid** | 33.84 | 29.7 | 38.53 | 49.38 | 26.6 | 32 | 22.27 | 20.94 | 15.05 | 17.1 | 26.46 | 21.26 | 18.66 | 17.95 | 18.77 | | 18.45 |  |

**Table M. Time to peak untwist rate**

|  | **Animal 1** | | **Animal 2** | | **Animal 3** | | **Animal 4** | | **Animal 5** | | **Animal 6** | | **Animal 7** | | **Animal 8** | |
| --- | --- | --- | --- | --- | --- | --- | --- | --- | --- | --- | --- | --- | --- | --- | --- | --- |
|  | **Test** | **Retest** | **Test** | **Retest** | **Test** | **Retest** | **Test** | **Retest** | **Test** | **Retest** | **Test** | **Retest** | **Test** | **Retest** | **Test** | **Retest** |
| **Base-Apex** | 290 | 320 | 290 | 290 | 270 | 300 | 320 | 200 | 375 | 265 | 260 | 265 | 325 | 305 | 210 | 270 |
| **Mid-Apex** | 335 | 320 | 290 | 290 | 205 | 300 | 275 | 200 | 315 | 265 | 260 | 265 | 285 | 300 | 210 | 375 |
| **Base-Mid** | 290 | 295 | 310 | 290 | 295 | 300 | 320 | 200 | 240 | 375 | 290 | 375 | 340 | 325 | 295 | 330 |

**Table N. Deceleration time**

|  | **Animal 1** | | **Animal 2** | | **Animal 3** | | **Animal 4** | | **Animal 5** | | **Animal 6** | | **Animal 7** | | **Animal 8** | |
| --- | --- | --- | --- | --- | --- | --- | --- | --- | --- | --- | --- | --- | --- | --- | --- | --- |
|  | **Test** | **Retest** | **Test** | **Retest** | **Test** | **Retest** | **Test** | **Retest** | **Test** | **Retest** | **Test** | **Retest** | **Test** | **Retest** | **Test** | **Retest** |
| **DT** | 53 | 49 | 55 | 75 | 80 | 80 | 70 | 83 | 55 | 50 | 75 | 85 | 55 | 75 | 80 | 97 |
